# Sibling species of the major malaria vector *Anopheles gambiae* display divergent preferences for aquatic breeding sites in southern Nigeria

**DOI:** 10.1186/s12936-024-04871-9

**Published:** 2024-02-27

**Authors:** Faith I. Ebhodaghe, Irma Sanchez-Vargas, Clement Isaac, Brian D. Foy, Elizabeth Hemming-Schroeder

**Affiliations:** 1 Center for Vector-Borne Infectious Diseases, Department of Microbiology, Immunology, and Pathology, Colorado State University, Fort Collins, CO USA; 2 Department of Zoology, Faculty of Life Sciences, Ambrose Alli University, Ekpoma, Edo State Nigeria

**Keywords:** *An. gambiae*, *An. coluzzii*, *An. arabiensis*, *An. stephensi*, Breeding behavior, Larval source management

## Abstract

**Background:**

When integrated with insecticide-treated bed nets, larval control of *Anopheles* mosquitoes could fast-track reductions in the incidence of human malaria. However, larval control interventions may deliver suboptimal outcomes where the preferred breeding places of mosquito vectors are not well known. This study investigated the breeding habitat choices of *Anopheles* mosquitoes in southern Nigeria. The objective was to identify priority sites for mosquito larval management in selected urban and periurban locations where malaria remains a public health burden.

**Methods:**

Mosquito larvae were collected in urban and periurban water bodies during the wet-dry season interface in Edo, Delta, and Anambra States. Field-collected larvae were identified based on PCR gel-electrophoresis and amplicon sequencing, while the associations between *Anopheles* larvae and the properties and locations of water bodies were assessed using a range of statistical methods.

**Results:**

Mosquito breeding sites were either man-made (72.09%) or natural (27.91%) and mostly drainages (48.84%) and puddles (25.58%). *Anopheles* larvae occurred in drainages, puddles, stream margins, and a concrete well, and were absent in drums, buckets, car tires, and a water-holding iron pan, all of which contained culicine larvae. Wild-caught *Anopheles* larvae comprised *Anopheles coluzzii* (80.51%)*, Anopheles gambiae *sensu stricto* (s.s.)* (11.54%)*,* and *Anopheles arabiensis* (7.95%); a species-specific PCR confirmed the absence of the invasive urban malaria vector *Anopheles stephensi* among field-collected larvae. *Anopheles arabiensis, An. coluzzii,* and *An. gambiae s.s*. displayed preferences for turbid, lowland, and partially sunlit water bodies, respectively. Furthermore, *An. arabiensis* preferred breeding sites located outside 500 m of households, whereas *An*. *gambiae s.s.* and *An*. *coluzzii* had increased detection odds in sites within 500 m of households. *Anopheles gambiae s.s.* and *An*. *coluzzii* were also more likely to be present in natural water bodies; meanwhile, 96.77% of *An. arabiensis* were in man-made water bodies. Intraspecific genetic variations were little in the dominant vector *An. coluzzii*, while breeding habitat choices of populations made no statistically significant contributions to these variations.

**Conclusion:**

Sibling malaria vectors in the *An. gambiae* complex display divergent preferences for aquatic breeding habitats in southern Nigeria. The findings are relevant for planning targeted larval control of *An. coluzzii* whose increasing evolutionary adaptations to urban ecologies are driving the proliferation of the mosquito, and *An. arabiensis* whose adults typically evade the effects of treated bed nets due to exophilic tendencies.

**Supplementary Information:**

The online version contains supplementary material available at 10.1186/s12936-024-04871-9.

## Background

Malaria remains a major public health challenge, with a disproportionately high burden of infections in Africa; ~ 95% of infection cases and ~ 96% of associated deaths are reported in the continent annually [1]. *Anopheles gambiae *sensu lato (*s.l.*) are the primary vectors of malaria in sub-Saharan Africa. These mosquitoes breed in clean and natural aquatic environments in the form of small sunlit water collections. However, deviations from this traditionally and widely known choice of breeding habitats by *An. gambiae s.l.* have been observed [2, 3], but these deviations remain understudied in countries in the West African sub-region. Furthermore, *An. gambiae s.l*. is a complex of mosquitoes comprising more than eight sibling species [4]. The anthropophilic and indoor-biting Afrotropical vectors *An. coluzzii* and *An. gambiae *sensu stricto (*s.s.*) —previously known as *M* and *S* molecular forms of *An. gambiae*, respectively— are sibling members of this complex and contribute to the high risk of malaria in Nigeria and neighbouring West and Central African countries [5].

Different mosquito larval surveys in West and Central Africa observed variations in the breeding habitat choices of *An. coluzzii* and *An. gambiae s.s*. (reviewed in [6]). In Burkina Faso, *An. coluzzii* co-existed with *An. gambiae s.s*. but preferred to breed in large, permanent, and vegetation-dense habitats (rice paddies), whereas *An. gambiae s.s*. preferred temporary puddles [7]. Additional differences in the ecologies of *An. coluzzii* and *An. gambiae s.s*. have been described in Mali [8] and Cameroon [9] among countries in the West and Central African sub-regions. Furthermore, evidence has emerged supporting the hypothesis that contrasting responses of larvae to breeding habitat conditions formed the basis for ecological speciation of *An. coluzzii* and *An. gambiae s.s*. [10]. However, underlying ecological factors that underpin oviposition site preferences of gravid females and the water properties that mediate segregation of the breeding habitats of *An. coluzzii* and *An. gambiae s.s*., are less well known. Addressing this knowledge gap, especially where *An. coluzzii* and *An. gambiae s.s*. are sympatric, is essential to reliably predict spatial distribution of larvae of the vector species and identify potential sites for targeted and species-specific mosquito larval control interventions.

Mosquito larval control interventions are effective for the control of malaria vectors. They also simultaneously target *Anopheles* and culicine disease vectors where these mosquitoes co-breed in water bodies [11]. Larval control interventions leverage biopesticides or predators to reduce the number of immature mosquitoes in aquatic environments and, where possible, may eliminate water bodies providing breeding places for mosquitoes [12]. However, mosquito larval control requires a clear understanding of the breeding habitats of target vectors in order to accurately select priority sites for interventions. Meanwhile, the World Health Organization [12] recommends mosquito larval control as a suitable method for supplementing pyrethroid-treated bed nets. This is because larval control reduces the abundance of pyrethroid-resistant mosquitoes, as well as outdoor-biting malaria vectors (e.g. *Anopheles arabiensis*) whose adults are typically outside the reach of pyrethroid-treated bed nets currently widely used in sub-Saharan African countries to control indoor-biting vectors.

Nigeria in West Africa reports the highest malaria disease burden worldwide, with > 25% of the global incidence of infections occurring in the country [1]. There are few reports on malaria spread by outdoor-biting mosquito species in Nigeria [13]. However, the typically indoor-biting vectors *An. coluzzii* and *An. gambiae s.s*. have been found to feed on humans in outdoor locations [14]. Some investigators attribute this to a behavioural response by vectors to the protracted use of pyrethroid-treated bed nets [15, 16]. Long-term adoption of treated bed nets has increased the frequencies of pyrethroid-resistant vectors in wild mosquito populations in southern Nigeria [17, 18], thus further compromising the efficacy of treated bed nets for malaria vector control. Although alternative intervention strategies, for example, mosquito larval control, are available for the management of malaria vectors, these strategies have received limited attention in southern Nigeria mainly due to the relatively low economic costs of using pyrethroid-treated bed nets.

Mosquito larval control interventions to manage pyrethroid-resistant *An. coluzzii* and *An. gambiae s.s*. could contribute to malaria risk reduction in southern Nigeria. These interventions could also assist in alleviating the epidemiologic burden of outdoor-biting *Anopheles* vectors that may be locally endemic but evading the effects of pyrethroid-treated bed nets. This study assessed the species diversity of *Anopheles* malaria vectors in selected urban and periurban areas in southern Nigeria. It further assessed vectors for differences in the choice of breeding habitats. Water bodies were surveyed for the presence and abundance of larvae and their physico-chemical properties characterised. Findings from the study add to current knowledge on the larval ecology of *An. gambiae s.l*. malaria vectors in Africa and provide relevant data for community-led and species-specific larval control interventions in urban and periurban settings in southern Nigeria where malaria risks are currently high and escalating.

## Methods

### Study area

Mosquito larval samplings were done in southern Nigeria with sites spread over a geographical distance of ≈200 km extending from Edo State (6° 17ʹ 1.341″ N, 5° 33ʹ 59.061″ E) to Delta State (6° 12ʹ 6.523″ N, 6° 10ʹ 47.316″ E) and Anambra State (6° 7ʹ 9.12″ N, 6° 47ʹ 15.792″ E). The human population size in these three neighboring States is ≈16 million [19]. According to the WHO Africa [19], household parasite screening surveys in 2021 based on the Rapid Diagnostic Technique (RDT) in children under 5 years of age indicated malaria infection rates of 30.2%, 18.9%, and 20.2% in Edo, Delta, and Anambra, respectively. Human exposure to infections is high in these areas during the wet season (May to mid-October), compared to the dry season (mid-October to April) when the numbers of vector breeding habitats are fewer. The average annual rainfall amount and temperature in southern Nigeria are 2500 mm and 27 ℃, respectively, while vegetation is typically rainforest with extensive networks of freshwater swamps, and sparse and scattered woodlands [20].

### Mosquito larvae sampling

Larval samplings were done during the late wet season and the early dry season from September to November 2022. To collect mosquito larvae in urban and periurban water bodies, a standard dipper (300 ml, John W. Hock’s Company, Gainesville, Florida, USA) was lowered towards a water body and carefully but quickly applied to scoop the water surface. Where present in a water sample, mosquito larvae were morphologically identified as either *Anopheles* or culicine, counted, and stored in alcohol within small, labeled vials. To estimate average larval abundance, the overall number of larvae collected in a water body was divided by the total number of dips made in the same water body.

### Characterization of water bodies

Water bodies within 500 m of households were considered ‘close’ while those outside 500 m were considered ‘far’. A water body was ‘turbid’ if it was difficult to clearly see through water sample and ‘non-turbid’ if otherwise. To determine the depth of mosquito larval sites, a straight pole was inserted in vertical position into a water body until the pole reached the bottom. Careful notice was made of the water-mark on the pole after it had been removed from water, while a graduated tape was used to measure the pole from the water mark down to the tip that touched the bottom of water body. Water bodies were considered ‘deep’ if they had a depth of above 20 cm and ‘shallow’ if depths were below 20 cm.

A handheld GPS device (Garmin etrex 10) was used to record geographic coordinates and altitude of sampling sites. Water samples were assessed for ‘temperature’, ‘pH’, and ‘salinity’ at each site using a calibrated multiparametric device (Hanna instrument GroLine Meter) powered by lithium batteries. Measurements of altitude (metres above sea level), temperature (℃), pH, and salinity (parts per million) were considered high if they exceeded the 65th percentile values of their respective distributions, otherwise they were low. The 65th percentile values for altitude, temperature, pH, and salinity were 136 m (62 m to 316 m, SD: ± 57.46), 30.03 °C (22.7 ℃ to 36.1 ℃, SD: ± 2.76), 7.74 (6.61 to 9.04, SD: ± 0.76), and 140 ppm (0 ppm to 410 ppm, SD: ± 101.79), respectively. Additional data collected at mosquito larval sites were the area (residential or industrial), site location (urban or periurban), habitat type (man-made or natural), vegetation presence (yes or no), presence of debris (yes or no), and water exposure to sunlight (partial or complete).

### Molecular identification of *Anopheles* larvae

Genomic DNA was extracted from each individual *Anopheles* larva following the Chelex protocol described by Musapa et al*.* [21]. The Polymerase Chain Reaction (PCR) gel electrophoresis method was used to identify *Anopheles* larvae and to differentiate species of *An. gambiae s.l.* by targeting the *S200 X6.1* insertion polymorphism present in *An. coluzzii* but absent in *An. gambiae s.s*., adopting the primers described by Santolamazza et al*.* [22] (Fwd: *TCGCCTTAGACCTTGCGTTA* and Rev: *CGCTTCAAGAATTCGAGATAC*). PCR was conducted in 12.5 µl reaction volume containing 1 µl template DNA, 0.25 µl (10 µM) of each primer, 4.75 µl nuclease-free water, and 6.25 µl OneTaq^®^ Quick-Load^®^ 2X Master Mix (New England Biolabs).

PCR for *Anopheles* DNA amplification targeting the *S200 X6.1* gene was carried out on a Thermal cycler (Eppendorf Mastercycler nexus gradient) at 94 ℃ for 30 s; 30 cycles of denaturation at 94 ℃ for 15 s, 54 ℃ for 30 s and 68 ℃ for 1 min, and a final extension at 68 ℃ for 5 min. *Anopheles* larvae were identified based on a base-pair size of ~ 479 *An. coluzzii* (*M* form) and ~ 249 for *An. gambiae s.s*. (*S* form). The species *An. gambiae s.s*. has similar base-pair sizes with its siblings namely *An. arabiensis, An. melas,* and *An. quadriannulatus*.

DNA amplifications of the *ITS2* gene were carried out to identify samples that failed to amplify the *S200 X6.1* gene using the primers ITS2A (Fwd: *TGTGAACTGCAGGACACAT*) and ITS2B (Rev: *TATGCTTAAATTCAGGGGGT*), with reaction volume as described for the *S200 X6.1* PCR above. PCR adopted reaction conditions similar to those described previously [23]. PCR cycling was carried out on a Thermal cycler (Eppendorf Mastercycler nexus gradient) at 95 ℃ for 3 min; 35 cycles of denaturation at 94 ℃ for 30 s, 55 ℃ for 30 s, and 72 ℃ for 45 s, and a final extension at 72 ℃ for 6 min. Band size for *Anopheles* using the *ITS2* gene marker was ~ 750 bp.

In an attempt to identify samples whose DNA failed to amplify in *S200 X6.1*-PCR and *ITS2*-PCR, an additional set of primers was used in endpoint-PCR namely, *St-F* (*CGTATCTTTCCTCGCATCCA*) targeting a region of the *ITS2* gene specific to *An. stephensi* and the universal primers *U5.8S-F* (*ATCACTCGGCTCATGGATCG*) and *UD2-R* (*GCACTATCAAGCAACACGACT*) [24]. PCR was carried out in 12.5 µl reaction volume containing 1 µl template DNA, 0.25 µl (10 µM) of each of the primers *St-F* and *U5.8S-F* and 0.4 µl (10 µM) of the primer *UD2-R*, 4.4 µl nuclease-free water, and 6.20 µl OneTaq^®^ Quick-Load^®^ 2X Master Mix (New England Biolabs). Cycling was carried out on a Thermal cycler (Eppendorf Mastercycler nexus gradient) at 95 ℃ for 30 s; 30 cycles of denaturation at 95 ℃ for 30 s, 55 ℃ for 30 s, and 68 ℃ for 45 s, and a final extension at 68 ℃ for 7 min. Band size for *An. stephensi* was ~438 bp with an internal control band of ~ 900 bp.

### Nucleotide sequencing and phylogenetic analysis

The species identities of *Anopheles* were confirmed by unidirectional sequencing at Azenta Life Sciences, Colorado State University, USA. Sequencing was carried out on cleaned PCR-products (Exo-CIP^™^, New England Biolabs) using the *S200 X6.1* primer sequence *TCGCCTTAGACCTTGCGTTA* [22] and the *ITS2B* primer sequence *TATGCTTAAATTCAGGGGGT* [23]. DNA sequences were visually inspected for quality in the *BioEdit* software [25]. Good-quality sequences were queried in BLAST analyses on the NCBI website [26]. Notes were taken of the sequence identities of query sequences compared to sequences of closest match in the GenBank. Clustal Omega [27] was used to align study and GenBank sequences, while the Smart Model Selection criterion in *PhyML* [28] was used to infer the best model of sequence evolution (Hasegawa-Kishino-Yano, HKY) [29]. Maximum-Likelihood phylogenetic trees were constructed in the software *Molecular Evolution and Genetic Analysis MEGA-X* [30], and the nodal support values of trees were estimated from 1000 bootstrap replications. Finally, genetic analyses to determine haplotype diversity (*Hd*) and polymorphic site number of *An. coluzzii* populations were carried out in *DnaSP* [31], while haplotype analyses were carried out using Median-Joining (MJ) networks [32]. MJ networks were constructed in the *PopART* software [33] with the aim to visualize relationships between populations of *An. coluzzii* larvae collected from water bodies in different geographical locations and having different properties.

### Data analyses

The numbers of: (i) water body sites with mosquito larvae, (ii) geo-referenced locations surveyed, and (iii) mosquito larval collections were expressed in percentage frequencies with 95% confidence intervals (CI). Differences between percentage frequencies were assessed using a two proportion *Z*-test.

To determine their relative importance, predictors of larvae presence in aquatic environments were ranked based on Random Forest (RF) classification analyses. RF analyses were based on 10,000 iterations (ntrees) with 4 variables randomly selected at each split (mtry = √q where q = the total number of variables (= 14)). The *R* functions ‘*importance* ()’ and ‘*varImpPlot* ()’ both embedded in the *randomForest* package version 4.7–1.1 [34] were used to generate Mean Decrease Gini (MDG) scores and variable importance plots, respectively. Variables with higher MDG scores were more important predictors of *Anopheles* larval presence in water bodies.

Generalized Linear Models (GLM) were used to assess associations between larvae and categorical predictor variables. Generalized Linear Models were fitted assuming a binomial distribution if response variable was binomial ("*larvae*_*yes*" or "*larvae*_*no*"), a negative binomial distribution if response variable was count (number of larvae per dip), and a quasi-binomial distribution if response variable was proportion (the number of larvae identified for a species divided by the total number of larvae analysed). A GLM assuming a binomial distribution was also used to assess the associations between *An. coluzzii* haplotypes and populations.

Variations in average larvae abundance were assessed using the Mann–Whitney *U* test. Correlations between larvae abundance and the following continuous variables (i) altitude, (ii) temperature, (iii) salinity, (iv) culicine abundance, and (v) pH were assessed in Spearman correlation tests and Principal Components Analysis (PCA). A Multiple Correspondence Analysis (MCA) was carried out to visually explore associations between larvae and water properties, as well as between larvae and location of mosquito breeding sites. PCA and MCA biplots were designed using the *R* packages *'FactorMineR'* and *'factorextra'* [35].

Multivariate Regression Models were fitted to account for possible confounding effects of variables. Predictor variables were selected for multivariate regression if they had *P* < 0.05 in univariate models. Furthermore, the backward elimination method was adopted to select predictor variables for the final multivariate model assuming a binomial distribution for binomial response variables, negative binomial distribution for count response variables, and quasi-binomial distribution for proportion response variables. Fisher’s Exact test was used to assess predictor variables for association. Associated predictor variables were mutually exclusive in the final model. Multivariate analyses were followed by pairwise comparisons with *Tukey*'s adjustment using the function ‘*emmeans’* embedded in the ‘*emmeans’* package [36]. All analyses were carried out in the *R* Statistical environment [37] while *P* values were set at an *alpha* of 0.05.

## Results

### Mosquito breeding habitats

**Fig. 1:**
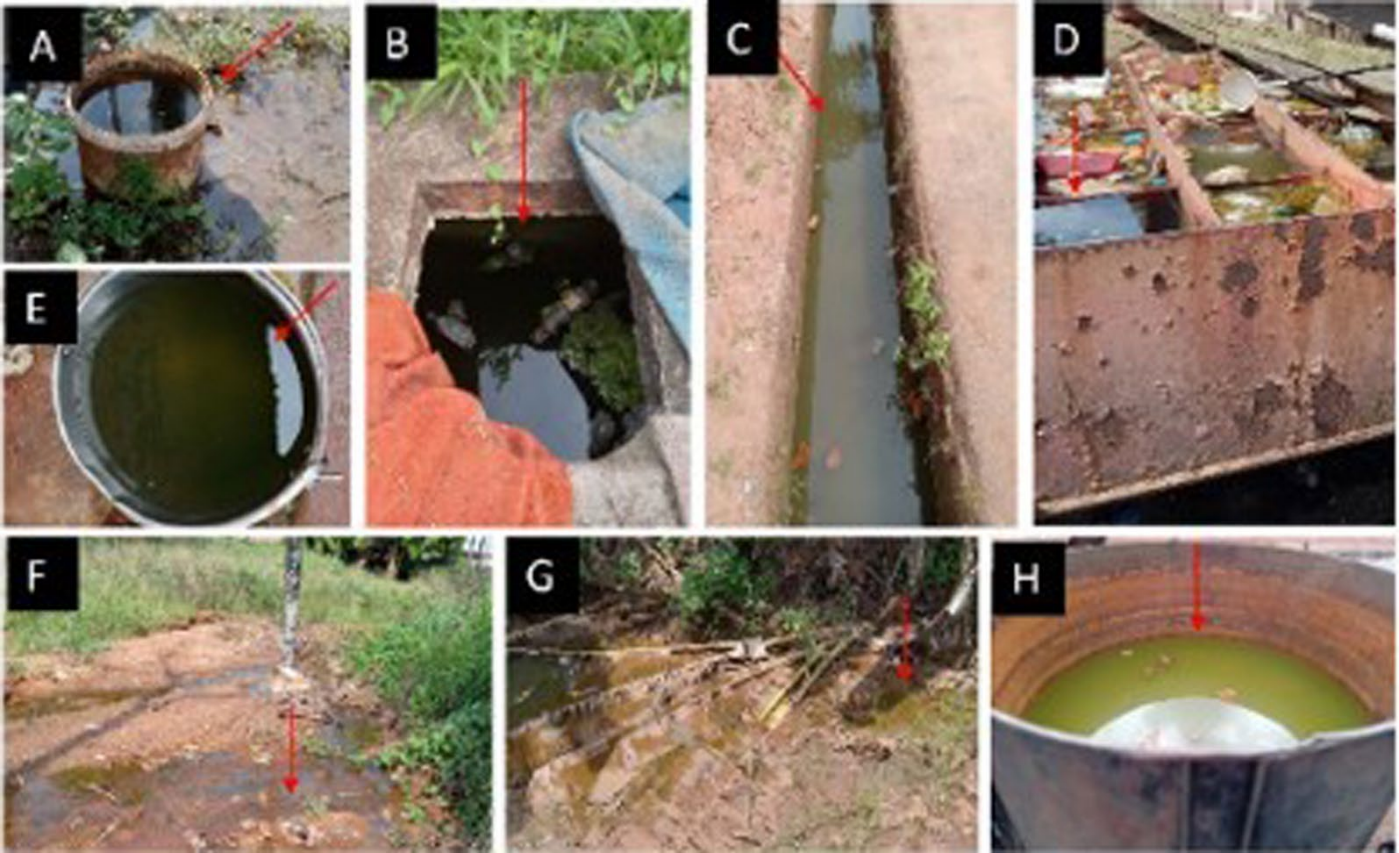
Mosquito breeding sites inspected for *Anopheles* larvae in southern Nigeria: **A** Plastic bucket, **B** concrete well, **C** drainage, **D** iron pan, **E** Aluminum bucket, **F** stream margin, **G** puddle, and **H** drum

**Fig. 2:**
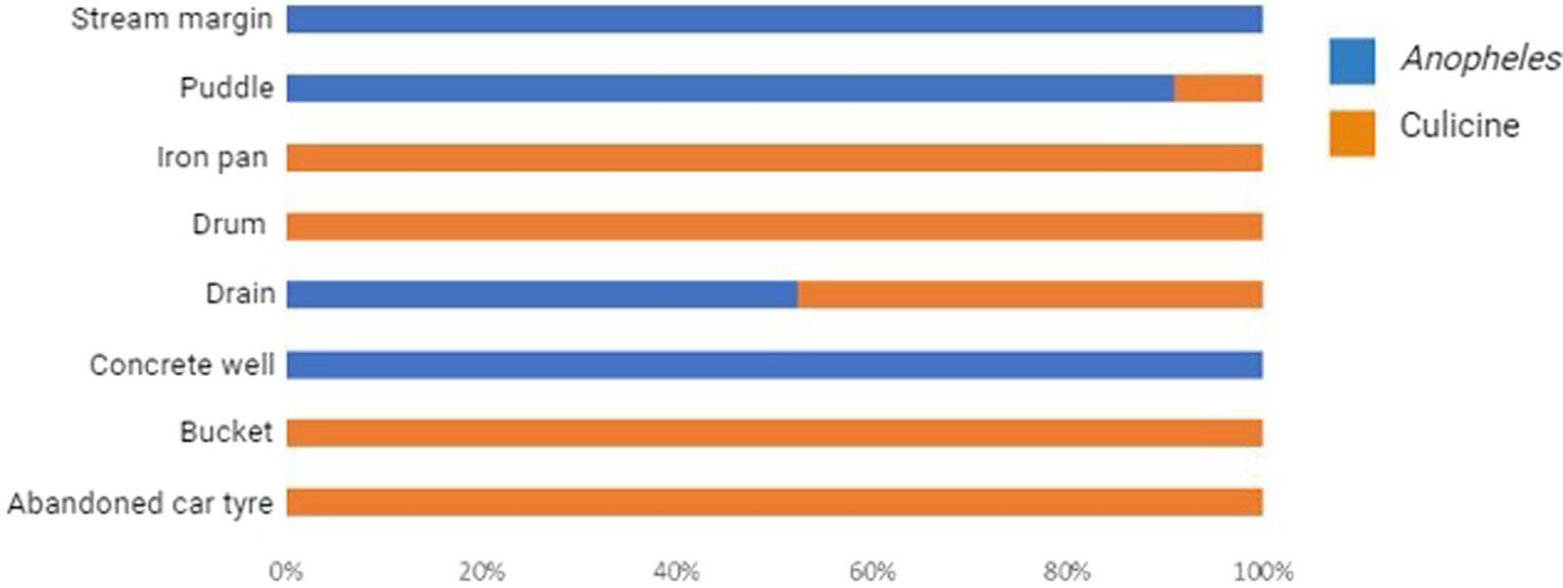
Proportions of *Anopheles* and culicine larvae in mosquito breeding sites in urban and periurban areas in southern Nigeria (September to November 2022)

A total of 43 water bodies among aquatic environments surveyed contained mosquito larvae. These water bodies were spread across 22 geo-referenced locations in Edo, Delta, and Anambra States (Table 1). These sites ranged from man-made (72.09%) to natural (27.91%) aquatic environments and comprised drains (48.84%), puddles (25.58%), abandoned car tires (9.30%), buckets (4.65%), drums (4.65%), and stream margins (2.33%), as well as iron pan (2.33%) and concrete well (2.33%) (Figs. 1 and 2). A total of 1,778 larvae collected comprised 32.34% *Anopheles* and 67.66% culicine mosquitoes. Additional file 1 shows the number of water bodies (according to habitat type) that were positive for *Anopheles* larvae and those that were positive for culicine larvae. Meanwhile, Additional file 2 provides an account of water properties identified to predict *Anopheles* larvae in water body sites.

**Table 1:** Geo-referenced locations of water bodies positive for mosquito larvae in urban and periurban areas in southern Nigeria (September to November 2022)

State	Location	Latitude	Longitude	Altitude	Mosquito larval habitat type
Edo	Aduwawa	6° 22′ 21.96″ N	5° 40′ 24.78″ E	106 m	Drain
	Ekenwan	6° 19′ 23.52″ N	5° 35′ 49.92″ E	87 m	Car tire, drain
	Ekiadolor*	6° 28′ 45.72″ N	5° 35′ 2.46″ E	136 m	Car tire, bucket, drum, iron pan
	GRA	6° 18′ 18.3″ N	5° 36′ 18.18″ E	66 m	Drain
	Ogbewase	6° 20′ 6.12″ N	5° 36′ 39.9″ E	132 m	Puddle
	Ogbeson	6° 20′ 32.49″ N	5° 41′ 11.68″ E	80 m	Drain, puddle
	Owina	6° 20′ 7.14″ N	5° 36′ 19.08″ E	94 m	Drain
	Sakponba	6° 18′ 47.94″ N	5° 38′ 6″ E	80 m	Car tire
	Ugbiyoko	6° 18′ 51.48″ N	5° 34′ 10.68″ E	77 m	Car tire, puddle
	Uwelu	6° 21′ 44.04″ N	5° 35′ 59.05″ E	92 m	Puddle
Delta	Agbor-Obi	6° 16′ 2.52″ N	6° 11′ 8.94″ E	131 m	Drain, drum
	Alihagwu*	6° 14′ 52.2″ N	6° 07′ 53.82″ E	168 m	Bucket
	Boji-Boji	6° 15′ 56.1″ N	6° 11′ 35.58″ E	116 m	Concrete well, drain, puddle
	Idumuoza*	6° 15′ 47.40″ N	6° 08′ 21.72″ E	169 m	Puddle
	Owa-Alero*	6° 12′ 32.46″ N	6° 13′ 20.64″ E	127 m	Puddle
	Owa-Eke*	6° 14′ 4.44″ N	6° 12′ 52.02″ E	203 m	Drain
	Umunede	6° 16′ 20.16″ N	6° 18′ 11.52″ E	252 m	Drain, puddle
Anambra	Ibolo-Oraifite*	6° 01′ 20.34″ N	6° 49′ 2.64″ E	62 m	Drain
	Nkpor*	6° 07′ 9.9″ N	6° 51′ 56.28″ E	113 m	Drain
	Nkwelle-Ezunaka*	6° 12′ 26.94″ N	6° 49′ 50.88″ E	77 m	Stream margin
	Odekpe*	6° 05′ 13.8″ N	6° 45′ 15.9″ E	64 m	Puddle
	Onitsha	6° 08′ 55.86″ N	6° 48′ 21.3″ E	110 m	Drain

^*^Peri-Urban

### Anopheles species diversity

*Sequencing and species identification* Overall, 528 out of the 575 field-collected *Anopheles* larvae were analyzed in PCR, with success of DNA amplification for 382 larvae in *S200 X6.1* gene-PCR and 42 larvae in *ITS2*-PCR; one larva was identified as *coluzzii-gambiae s.s.* hybrid in the *S200 X6.1* gene-PCR. The remaining 104 samples that failed to amplify in *S200 X6.1* gene-PCR and *ITS2*-PCR were analysed using molecular markers that target amplification of *An. stephensi* DNA; however, none of these 104 samples amplified in endpoint-PCR except for the *An. stephensi* positive control included in the reaction. Further, 78 samples randomly selected from the 382 samples that amplified in *S200 X6.1* gene-PCR and all 42 samples that amplified in *ITS2*-PCR were submitted to Sanger sequencing in order to confirm species identity. For *S200 X6.1* sequences, NCBI BLAST search identified 58 sequences as *An. coluzzii*, 4 sequences as *An. gambiae s.s*., and 6 sequences as *An. arabiensis,* while 10 sequences had poor quality and were thus excluded from further analysis. For *ITS2* sequences, 7 sequences having poor quality were discarded, while 3 sample sequences were identified in NCBI BLAST analysis as *An. arabiensis* and 32 sample sequences were identified as *An. gambiae s.l*.

*Agreements between PCR-gel electrophoresis and amplicon sequencing for Anopheles species identification* *S200 X6.1* PCR and amplicon sequencing had near perfect agreement for the identification of *An. coluzzii* (Cohen’s Kappa K = 0.84) and *An. gambiae s.s*. (Cohen’s Kappa K = 0.90), but no agreement for the identification of *An. arabiensis* (Cohen’s Kappa K = 0.00). The lack of agreement between *S200 X6.1* PCR and amplicon sequencing for *An. arabiensis* identification was due to the similarity of band sizes between *An. gambiae ss* (~ 249 bp) and *An. arabiensis* (~ 223 bp). The similarity resulted in *An. arabiensis* mis-identification as *An. gambiae s.s*. in endpoint-PCR, but this mis-identification was corrected in amplicon sequencing. Six (6) *An. gambiae s.s*. samples and 2 *An. coluzzii* samples so identified by PCR were shown by sequencing to be *An. arabiensis* and *An. gambiae s.s*., respectively. In an attempt to ensure that the study did not miss out on *An. arabiensis,* samples identified in endpoint PCR as *An. gambiae s.s*. were selected from different sites for amplicon sequencing.

*Percentage identities and DNA sequence lengths* Percentage identities of DNA sequences from the study when compared to GenBank DNA sequences ranged between 99.26% and 100% for the *S200 X6.1* sequences with base-pair (bp) lengths of between 171bp and 180bp for *An. arabiensis*, 194bp and 210bp for *An. gambiae s.s*., and 407bp to 430bp for *An. coluzzii*. For *ITS2* sequences, percentage identities ranged between 99.5% and 100% with base-pair lengths of between 508bp and 516bp for *An. arabiensis* and 399bp and 531bp for *An. gambiae s.l*.

**Fig. 3:**
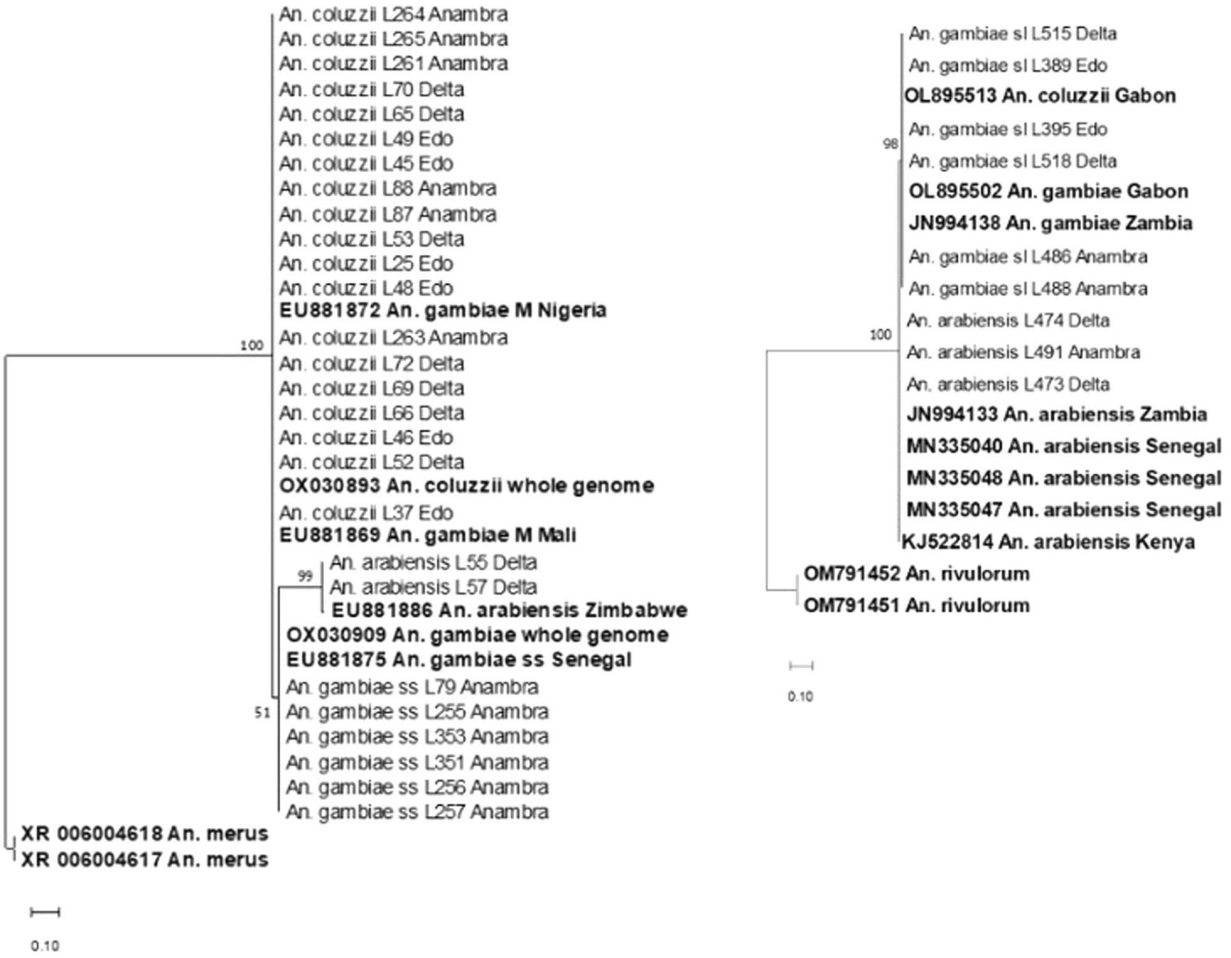
*S200x6.1* DNA-based (left) and *ITS2* DNA-based (right) Maximum-Likelihood phylogenetic trees. Each tree shows the phylogenetic relationships between *Anopheles* sample collected in southern Nigeria (September to November 2022). DNA sequences from this study end with the name of the state (Edo, Delta, or Anambra) in southern Nigeria where samples were collected, while sequences from GenBank are shown in bold. GenBank sequence of *An. merus* and *An. rivulorrum* have been selected as outgroup respectively for the *S200x6.1* DNA-based phylogeny (left) and *ITS2* DNA-based phylogeny (right). Nodal support values based on 1000 bootstrap replicates are indicated next to the relevant nodes. The branch length represents substitution per site

*S200 X6.1 phylogeny* Study DNA sequences of *An. coluzzii* on a maximum-likelihood phylogenetic tree (Fig. 3) clustered with GenBank DNA sequences of *An. coluzzii* whole genome (Accession No.: OX030893) and sequences of *An. gambiae* M molecular form from Mali (Accession No.: EU881869) and Nigeria (Accession No.: EU881872). Further, *An. gambiae s.s*. study sequences clustered with GenBank sequences of the whole genome of *An. gambiae s.s*. (Accession No.: OX030909) and a *An. gambiae s.s*. sequence from Senegal (Accession No.: EU881875). Lastly, DNA sequences of *An. arabiensis* from the study clustered with a sequence of the same species from Zimbabwe (Accession No.: EU881886).

*ITS2 phylogeny *On the maximum-likelihood phylogenetic tree constructed from *ITS2* sequences (Fig. 3), *An. arabiensis* study sequences clustered with *An. arabiensis* GenBank sequences from Senegal (Accession Nos. MN335047, MN335048, and MN335040), Kenya (Accession No.: KJ522814) and Zambia (Accession No.: JN994133), whereas study sequences of *An. gambiae s.l.* clustered with GenBank sequences of *An. coluzzii* from Gabon (Accession No.: OL895513) and *An. gambiae* from Gabon (Accession No. OL895502) and Zambia (Accession No. JN994138).

### *Anophelescoluzzii*, *An. gambiae s.s*., and *An. arabiensis*

**Fig. 4:**
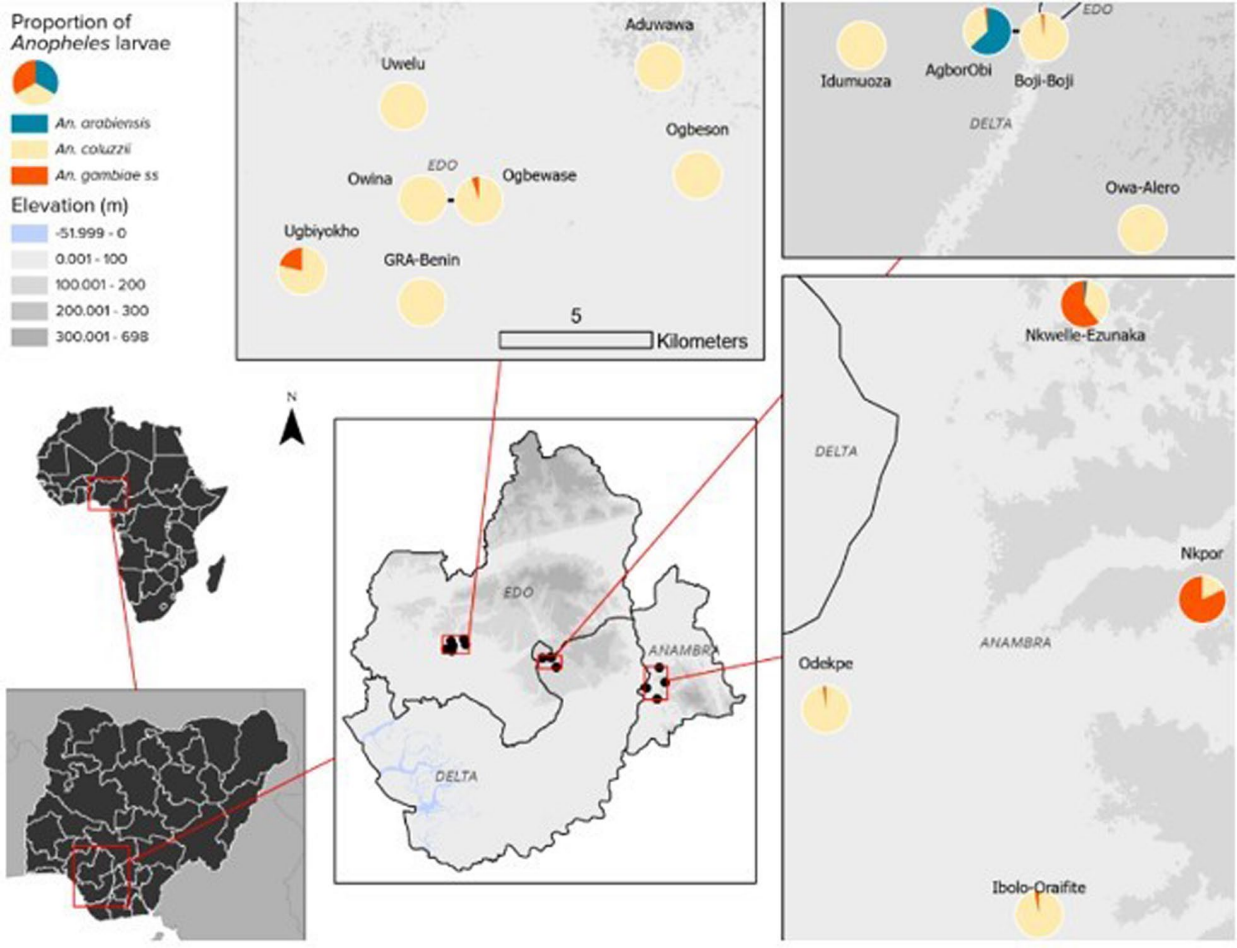
Map of sampling locations in southern Nigeria showing the relative proportions of *An. gambiae sl* larvae

**Fig. 5:**
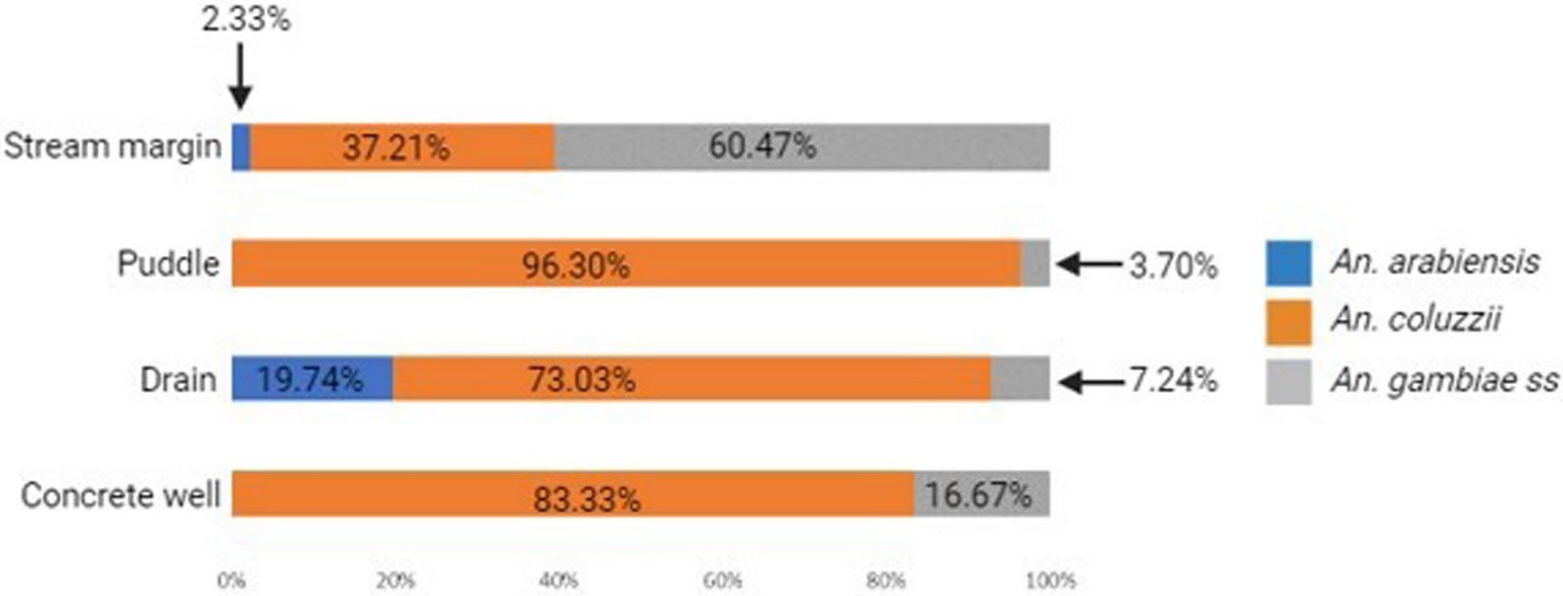
Relative proportions of sibling species of *An. gambiae sl* in different breeding habitat types in southern Nigeria

*Overall distribution and proportions* *An. coluzzii* occurred at more locations (68.18%, 15/22) compared to *An. arabiensis* (9.09%, 2/22) (Z-test: *P* = 0.0002). However, the number of occurrence locations were similar between *An. coluzzii* and *An. gambiae s.s*. (36.36%, 8/22) (*P* = 0.07) and between *An. arabiensis* and *An. gambiae s.s*. (*P* = 0.07) (Fig. 4, Additional file 3). Overall, *An. coluzzii* larvae represented a greater proportion (80.51%, 314/390) in comparison to *An. gambiae s.s*. (11.54%, 45/390) (Z-test: *P* < 0.0001) and *An. arabiensis* (7.95%, 31/390) (*P* < 0.0001), whereas *An. gambiae s.s*. and *An. arabiensis* occurred at similar proportions (*P* = 0.1165). *Anopheles coluzzii* were detected mainly in puddles (57.96%, 182/314), and then in drains (35.35%, 111/314), stream margin (5.10%, 16/314), and a concrete well (1.596%, 5/314), while for *An. gambiae s.s*., detections were mainly in stream margin (57.78%, 26/45), followed by drains (24.44%, 11/45), puddles (15.56%, 7/45), and a concrete well (2.22%, 1/45) (Fig. 5). *Anopheles arabiensis* were detected at stream margin (3.23%, 1/31) and mainly in drains (96.77%, 30/31). *Anopheles coluzzii* and *An. gambiae s.s*. co-existed at 4 puddle sites and 3 drain sites, as well as in a concrete well. Meanwhile, *An. coluzzii* co-existed with *An. arabiensis* in drains at 2 sites, whereas all three species co-existed at stream margin.

**Fig. 6:**
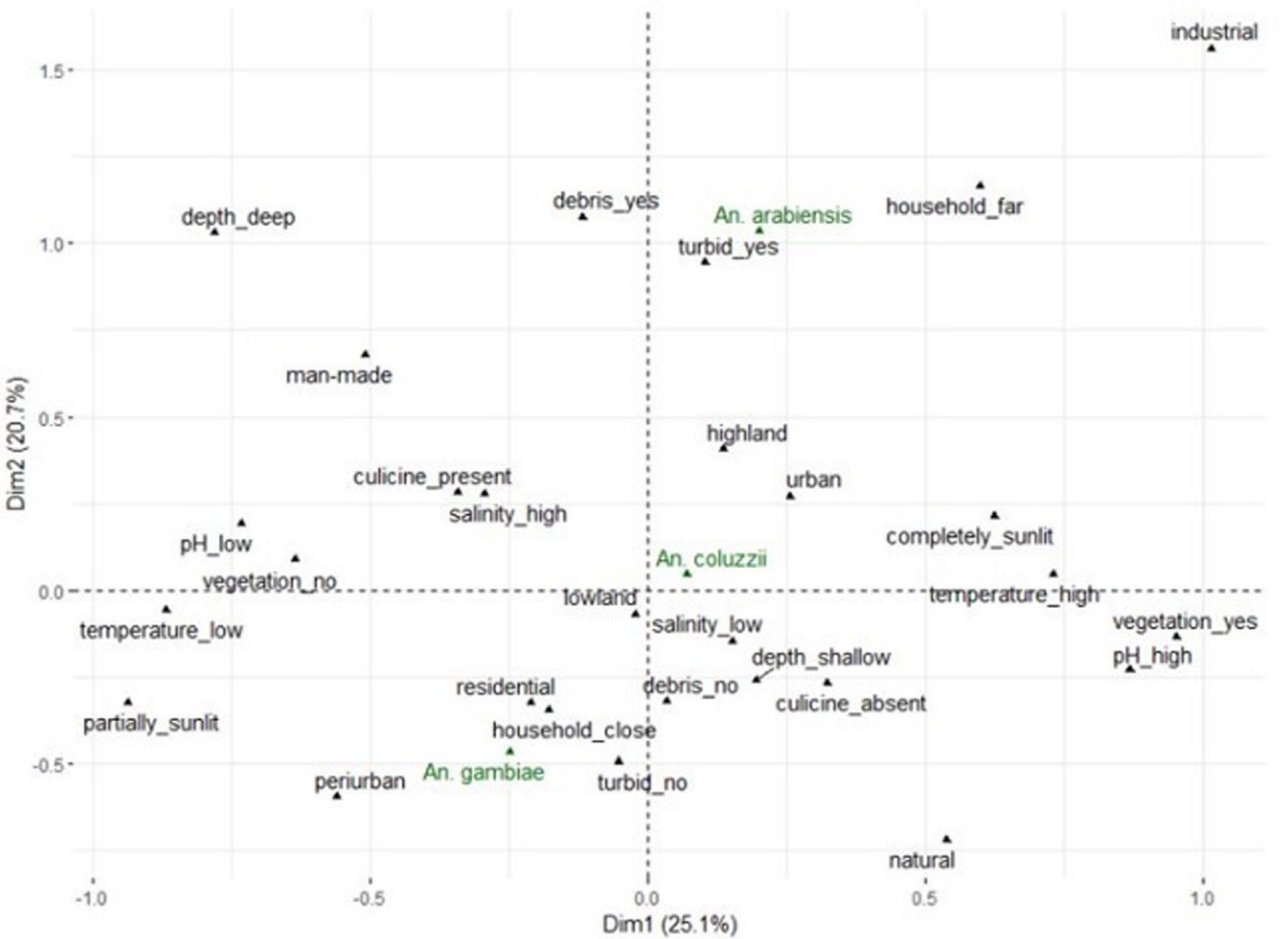
A multiple correspondences analysis (MCA) biplot to visually illustrate the association between sibling malaria vectors and water properties as well as locations of mosquito breeding sites in southern Nigeria (September to November 2022)

*Multiple correspondence analysis* The first two dimensions of the MCA explained 45.8% of variations in the properties of larval microhabitats (Fig. 6). Twenty-five percent and 20.7% of these variations were respectively accounted for by dimensions 1 and 2. Sibling species of *An. gambiae s.l*. separated more clearly along dimension 2 than dimension 1. Among water properties, water turbidity and the presence of debris in water made greater contributions to variations on dimension 2. The vector *An. gambiae s.s.* occupied the negative axis of dimension 2, whereas *An. coluzzii* and *An. arabiensis* occupied the positive axis of the same dimension (Fig. 6).

Separation of sibling species on the MCA biplot was more apparent for *An. gambiae s.s*. and *An. arabiensis* than for any other pair of the sibling vectors (Fig. 6). On dimension 2 of the biplot and more than the other vectors, *An. gambiae s.s*. showed close association with non-turbid water bodies and aquatic environments without debris and with partial exposure to sunlight, while also having closer association with human dwellings and periurban locations. Whereas the association of *An. coluzzii* with water properties on dimension 2 was less clear, *An. arabiensis* displayed close associations with turbid water, as well as water bodies containing debris and those far from households.

*Random Forest classification* On variable importance plots (Additional file 4), presence of culicine mosquitoes and debris, and habitat type and altitude were stronger predictors of *Anopheles* larvae and *An. coluzzii* presence (RF Accuracy 72.09%), whereas presence of *An. gambiae s.s*. depended more on turbidity, exposure to sunlight, and presence of culicine mosquitoes (RF Accuracy 83.72%) and *An. arabiensis* depended on culicine presence, temperature, and location (residential vs industrial) (RF Accuracy 93.02%).

*Odds ratio analysis* The odds of *An. coluzzii* detection were greater in natural habitats (OR: 17.42, 95% CI 2.85, 339.61) and water bodies without debris (OR: 5.40, 95% CI 1.49, 22.22). These odds were also greater in lowlands (OR: 4.74, 95% CI 1.25, 21.13) and in aquatic environments with relatively high temperatures (OR: 6.18, 95% CI 1.55, 31.95) and pH (OR: 5.19, 95% CI 1.30, 26.80) (Table 2).

**Table 2:** The odds ratio of various predictor variables for the sibling species *An. coluzzii, An. gambiae ss,* and *An. arabiensis* collected in urban and periurban areas in southern Nigeria (September to November 2022)

Predictor variables		Odds ratio (95% CI) (*P*-value, binomial GLM)					
		*An. coluzzii*	*P* value	*An. gambiae ss*	*P* value	*An. arabiensis*	*P* value
Culicine presence	Yes	NA	NA	0.14 (0.04, 0.36)	**0.029**	NA	NA
	No			5.83 (1.22, 30.75)			
Household	Far	0.63 (0.19, 1.87)	0.2	0.08 (0.00, 0.42)	0.19	0.18 (0.03, 0.68)	0.19
	Close	2.40 (0.64, 9.68)		4.36 (0.68, 85.97)		0.19 (0.01, 2.17)	
Area	Industrial	0.67 (0.17, 2.33)	0.33	NA	NA	0.25 (0.04, 1.00)	0.11
	Residential	2.04 (0.49, 9.29)				0.13 (0.01, 1.46)	
Location	Periurban	1.00 (0.31, 3.20)	0.78	0.50 (0.13, 1.59)	0.22	0.09 (0.00, 0.47)	0.83
	Urban	1.21 (0.31, 4.72)		0.38 (0.08, 1.87)		0.76 (0.07, 17.29)	
Altitude	Highland	0.40 (0.11, 1.20)	**0.028**	0.08 (0.00, 0.39)	0.15	NA	NA
	Lowland	4.75 (1.25, 21.13)		4.95 (0.78, 97.40)			
Habitat	Man-made	0.63 (0.30, 1.29)	**0.0099**	0.15 (0.04, 0.38)	**0.047**	0.07 (0.01, 0.23)	0.83
	Natural	17.42 (2.85, 339.61)		4.82 (1.03, 24.60)		1.32 (0.06, 15.16)	
Turbidity	Yes	1.00 (0.39, 2.56)	0.7	0.06 (0.00, 0.29)	0.062	0.13 (0.02, 0.44)	0.39
	No	1.27 (0.38, 4.36)		8.00 (1.27, 156.90)		0.33 (0.01, 3.76)	
Debris	Yes	0.42 (0.13, 1.12)	**0.013**	0.06 (0.00, 0.31)	0.078	0.13 (0.02, 0.47)	0.34
	No	5.40 (1.49, 22.22)		7.11 (1.12, 139.48)		0.30 (0.01, 3.39)	
Vegetation	No	0.87 (0.41, 1.82)	0.21	0.27 (0.10, 0.63)	0.91	0.08 (0.01, 0.26)	0.95
	Yes	2.31 (0.64, 9.09)		0.92 (0.17, 4.17)		0.93 (0.04, 10.54)	
Depth	Deep	1.00 (0.24, 4.23)	0.83	0.33 (0.05, 1.45)	0.75	0.14 (0.01, 0.80)	0.51
	Shallow	1.19 (0.24, 5.77)		0.75 (0.14, 5.86)		0.42 (0.04, 9.87)	
pH	Low	0.71 (0.33, 1.47)	**0.029**	0.21 (0.07, 0.50)	0.4	0.07 (0.01, 0.25)	0.98
	High	5.19 (1.30, 26.80)		1.92 (0.40, 8.82)		1.04 (0.05, 11.83)	
Salinity	Low	1.00 (0.49, 2.06)	0.49	0.25 (0.09, 0.57)	0.82	0.07 (0.01, 0.24)	0.9
	High	1.60 (0.43, 6.38)		1.20 (0.22, 5.57)		1.17 (0.05, 13.35)	
Temperature	Low	0.65 (0.29, 1.37)	**0.016**	0.17 (0.05, 0.43)	0.15	0.04 (0.00, 0.17)	0.26
	High	6.18 (1.55, 31.95)		3.00 (0.66, 14.50)		4.15 (0.37, 94.32)	
Exposure to sunlight	Complete	1.23 (0.59, 2.60)	0.75	0.12 (0.03, 0.33)	**0.022**	0.07 (0.01, 0.25)	0.98
	Partial	0.81 (0.22, 2.95)		6.50 (1.40, 36.90)		1.04 (0.05, 11.83)	

*NA* Not Available due to small sample size. *P* values <0.05 are shown in bold.

Odds of *An. gambiae s.s*. detection were greater in water bodies with partial rather than complete exposure to sunlight (OR: 6.50, 95% CI 1.40, 36.90), as well as in natural habitats (OR: 4.82, 95% CI 1.03, 24.60) and water bodies without culicine mosquitoes (OR: 5.83, 95% CI 1.22, 30.75) (Table 2).

Based on multivariate binomial regression modeling, topographic altitude and habitat exposure to sunlight respectively predicted the presence of *An. coluzzii* and *An. gambiae s.s*. in water bodies (Additional file 5). Habitat type also predicted the presence of *An coluzzii* and *An. gambiae s.s*. in the multivariate model analysis. The presence of *An. arabiensis* in water bodies was not assessed in multivariate regression due to small sample size.

*Mean proportions* The relative mean proportion of *An. coluzzii* was greater in natural habitats (64.76, 95% CI 45.24, 84.27) and debris-free water bodies (46.96, 95% CI 31.58, 62.35) (Table 3). *Anopheles gambiae s.s*. also occurred at greater proportion in debris-free water bodies (6.05, 95% CI 0.45, 11.65), as well as in water bodies that are non-turbid (5.93, 95% CI 0.13, 11.73) and partially sunlit (9.50, 95% CI 0.00, 19.83) (Table 3).

**Table 3:** The proportions of sibling malaria vectors in mosquito breeding sites in urban and periurban areas in southern Nigeria (September to November 2022)

Predictor variables		Mean proportion (95% CI) (*P-*value, Mann–Whitney *U* test)					
		*An. coluzzii*	*P* value	*An. gambiae ss*	*P* value	*An. arabiensis*	*P* value
Culicine abundance	High	22.41 (1.53, 43.28)	0.116	4.86 (0.00, 13.09)	0.519	NA	
	Low	40.95 (26.14, 55.77)		3.14 (0.00, 6.52)		6.34 (0.00, 15.28)	
Household	Close	41.84 (27.65, 56.04)	0.1357	3.48 (0.20, 6.76)	0.227	0.05 (0.00, 0.17)	0.151
	Far	17.49 (0.00, 39.12)		4.33 (0.00, 13.75)		13.54 (0.00, 33.50)	
Area	Residential	38.42 (24.99, 51.85)	0.3473	4.87 (0.44, 9.30)		0.05 (0.00, 0.15)	0.064
	Industrial	21.49 (0.00, 50.28)		NA		17.60 (0.00, 44.13)	
Location	Urban	35.14 (21.27, 49.00)	0.9772	1.84 (0.10, 3.57)	0.216	5.68 (0.00, 13.74)	0.902
	Periurban	32.80 (5.88, 59.71)		8.65 (0.00, 20.91)		0.14 (0.00, 0.44)	
Altitude	Lowland	39.32 (25.11, 53.53)	0.1293	5.11 (0.10, 10.12)	0.135	6.12 (0.00, 14.75)	
	Highland	24.46 (0.97, 47.95)		0.89 (0.00, 2.82)		NA	
Habitat	Natural	64.76 (45.24, 84.27)	**0.001**	6.00 (0.00, 13.96)	0.062	0.14 (0.00, 044)	0.902
	Man-made	22.76 (9.88, 35.65)		2.86 (0.00, 6.74)		5.68 (0.00, 13.74)	
Turbidity	Non-turbid	38.31 (21.88, 54.74)	0.5079	5.93 (0.13, 11.73)	**0.041**	0.07 (0.00, 0.20)	0.358
	Turbid	29.16 (10.57, 47.76)		0.69 (0.00, 2.16)		9.78 (0.00, 23.92)	
Debris	No	46.96 (31.58, 62.35)	**0.012**	6.05 (0.45, 11.65)	**0.049**	0.06 (0.00, 0.19)	0.311
	Yes	15.40 (0.00, 31.73)		0.20 (0.00, 0.61)		10.35 (0.00, 25.37)	
Vegetation	Yes	47.37 (24.19, 70.55)	0.104	1.85 (0.00, 4.36)	0.844	5.90 (0.00, 18.55)	1.000
	No	27.58 (13.76, 41.40)		4.75 (0.00, 9.92)		3.18 (0.00, 9.59)	
Depth	Shallow	35.10 (21.80, 48.41)	0.869	3.76 (0.00, 7.86)	0.709	2.57 (0.00, 7.71)	0.525
	Deep	31.77 (0.00, 65.79)		3.65 (0.00, 9.37)		10.94 (0.00, 36.80)	
pH	High	50.01 (29.77, 70.25)	0.071	2.10 (0.00, 4.78)	0.572	6.32 (0.00, 19.97)	0.953
	Low	26.99 (12.31, 41.66)		4.53 (0.00, 9.53)		3.07 (0.00, 9.25)	
Salinity	High	40.98 (16.56, 65.40)	0.494	1.66 (0.00, 4.45)	0.956	6.80 (0.00, 21.63)	0.881
	Low	31.67 (17.41, 45.92)		4.64 (0.00, 9.46)		2.97 (0.00, 8.93)	
Temperature	High	46.52 (25.30, 67.75)	0.053	5.71 (0.00, 13.84)	0.191	11.73 (0.00, 28.87)	0.226
	Low	28.03 (13.34, 42.72)		2.68 (0.00, 6.07)		0.06 (0.00, 0.18)	
Exposure to sunlight	Partial	32.28 (9.28, 55.28)	0.848	9.50 (0.00, 19.83)	**0.013**	0.12 (0.00, 0.37)	1.000
	Complete	35.55 (20.83, 50.27)		0.96 (0.00, 2.23)		6.07 (0.00, 14.70)	

*NA* Not Available due to small sample size.  *P* values <0.05 are shown in bold.

**Fig. 7:**
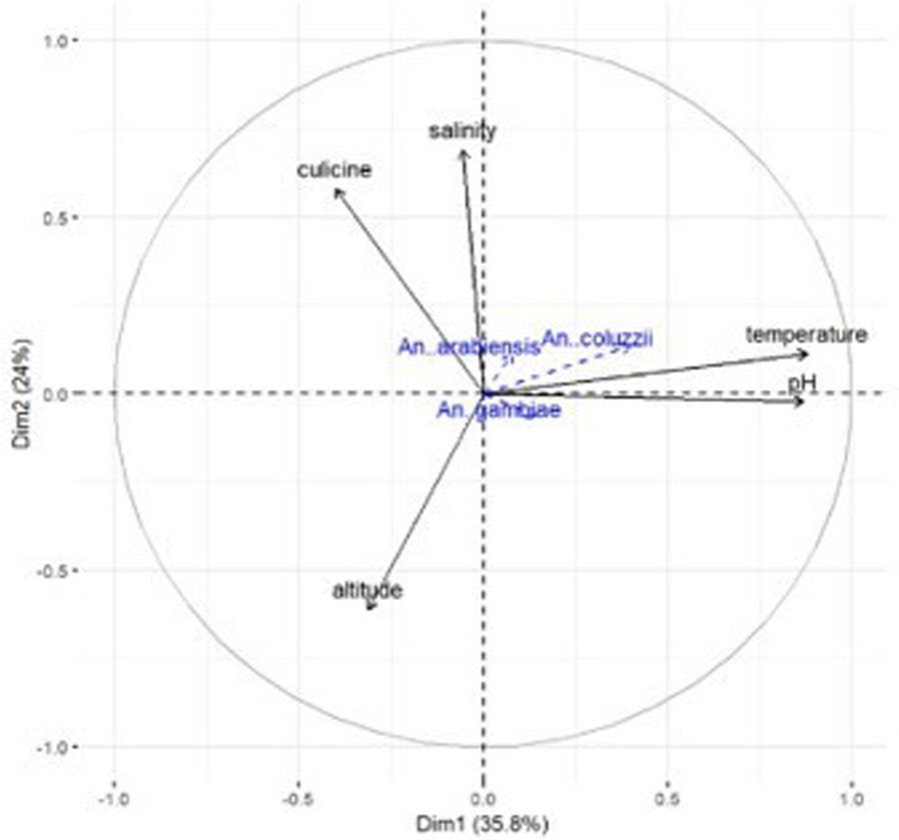
A principal component analysis (PCA) biplot to visually illustrate the association of sibling malaria vectors with topographic altitude and water properties of mosquito breeding sites

Proportions of *An. coluzzii* were negatively associated with altitude (ρ = − 0.33, P = 0.033) and positively associated with temperature (ρ = 0.36, P = 0.019). Figure 7 shows the direction and magnitude of these associations. Among predictor variables, only habitat exposure to sunlight could predict the proportions of *An. gambiae s.s*. in multivariate quasibinomial regression models (Additional file 5). No predictor variable was identified to statistically predict the proportions of *An. coluzzii* in multivariate models. The association between predictor variables and proportions of *An. arabiensis* could not be assessed in multivariate regression due to the small sample size.

### Haplotype diversity of *An. coluzzii* populations based on DNA sequences of the *S200 X6.1* gene

A total of 58 *An. coluzzii* DNA sequences (391 bp) were analysed to assess possible effects of breeding habitat choices on genetic variations in *An. coluzzii* populations. The DNA sequences of *An. coluzzii* consisted of 6 haplotypes (*H1* to *H6*), with a diversity (*Hd*) of 0.62 (Additional file 6). These haplotypes segregated at 5 polymorphic sites, in addition to one nucleotide deletion in one of the study sequences (GenBank Accession Number: OR700036).

Additional file 7 shows the nucleotide positions of these segregations with reference to the *An. coluzzii* Ngousso genome hosted in VectorBase [38]. The nucleotide substitution A > G was the most common mutation while the nucleotide substitution A > C was the least common mutation. The *H1* haplotype was more likely to occur in urban locations (OR: 3.80, 95% CI 1.21, 13.62) than periurban locations (OR: 0.29, 95% CI 0.10, 0.74) (*P* = 0.028). Aside this, no other association was detected between haplotype and geographical location or property of water bodies. Further analysis revealed low genetic variations between *An. coluzzii* populations that selected different breeding sites (Additional file 8).

## Discussion

This study characterized the species diversity and breeding habitat choices of *Anopheles* malaria vectors in selected urban and periurban areas in southern Nigeria. Wild-caught *Anopheles* mosquito larvae comprised *An. coluzzii*, *An. gambiae s.s*., and *An. arabiensis* with preferred breeding sites in lowland, partially sunlit, and turbid water bodies, respectively. Furthermore, *An. coluzzii* and *An. gambiae s.s*. showed close association with breeding sites within 500 m of households, whereas *An. arabiensis* were associated with breeding sites outside 500 m of households.

Similar to findings in Ghana’s Cape Coast in West Africa [2], *An. coluzzii* occurred over a wider spatial range and at higher proportions compared to other sibling species. This may be explained by the adaptation of *An. coluzzii* larvae to predator pressure in the wild and the vector's ability to outcompete sibling species in natural environments [39]. Most mosquito breeding grounds in southern Nigeria had dried up during the dry season campaign. This left behind permanent breeding grounds that favour the proliferation of *An. coluzzii* and, as in a previous work [7], contributed to increase collection of this vector species.

The overexpression of detoxification genes by *An. coluzzii* has been demonstrated to enable larval individuals to exploit polluted urban breeding sites in Central Africa [40] and may further explain the high odds of *An. coluzzii* in urban and periurban water bodies in southern Nigeria. Findings revealed that *An. coluzzii* larvae were more likely to occur in water bodies in lowlands than in highlands. Except for a few cases where broad flat surfaces provided breeding places for mosquitoes in highlands [41], water bodies are more stable for mosquito breeding activities in lowlands. Also, warm temperatures in lowlands are favourable for mosquito larval survival and assist to accelerate rates of larval development [42–44].

Even though sampling did not include adult mosquitoes, it is likely that *An. coluzzii* dominated adult populations of malaria vectors during the predominantly dry sampling period in southern Nigeria and that this contributed to increase collections of *An. coluzzii* larvae in study locations. Interestingly, earlier studies in similar ecologies that were conducted during dry periods of the year when temperatures are high alluded to the dominance of *An. coluzzii* among adult *Anopheles* mosquitoes [45, 46]. Under high temperature conditions, adult *An. coluzzii* are more able than other sibling vectors to minimize water loss and they do this by a variety of methods including altering the chemical compositions of cuticular hydrocarbons [47].

*Anopheles gambiae s.s*. have the behaviour of ovipositing in temporary, rain-dependent, small water collections such as puddles and hoof-prints [6]. As evaporation of small water collections is likely to occur over shorter periods during dry seasons, it seems that *An. gambiae s.s*. in southern Nigeria have developed a strategy that allows more time to complete development of immature stages before breeding habitats completely dry up. This strategy, as the study results suggest, involves *An. gambiae s.s*. preference for water collections that are partially rather than completely exposed to sunlight. The present study therefore hypothesizes that direct and complete exposure to sunlight could hasten evaporation of temporary water bodies where *An. gambiae s.s*. breed and result in the death of immature mosquitoes before they reach adulthood.

For multiple reasons, wild-caught larvae in Delta and Anambra were not expected to include the outdoor-biting mosquito *An. arabiensis*. Firstly, *An. arabiensis* typically inhabit arid savannah landscapes and are often absent in field collections of adult or larval mosquitoes in the humid rainforest zone of southern Nigeria [48]. Secondly, except on very few occasions [49–51], past and recent surveys in Delta and Anambra States have failed to detect *An. arabiensis* in field campaigns [14, 17, 52]. Possible reasons for the absence of *An. arabiensis* in these earlier campaigns include but are not limited to the possibility that the vector was simply not present in sampling areas, or that investigators focused samplings on adult mosquitoes indoors and identified mosquitoes using less-sensitive techniques that are incapable of teasing apart sibling species.

In separate studies in Burkina Faso in West Africa [7] and Kenya in East Africa [53], *An. arabiensis* had its highest abundance during the dry season in October about the same time when *An. gambiae s.s*. had its lowest abundance. Mosquito larval collection during a similar period of the year likely increased the chances of *An. arabiensis* detection. This mosquito species is zoophilic and occurs close to livestock [54], thus it is not surprising that mosquito sampling led to collection of many *An. arabiensis* larvae in water bodies in Agbor-Obi (Delta State) where there are several pockets of livestock-keeping areas.

The association between *An. arabiensis* and livestock production has been confirmed in a plethora of studies in sub-Saharan Africa [55–57] and is based on the fact that female mosquitoes, usually, restrict flight activities to places near animal blood meal hosts and oviposit in water bodies nearby. This could also explain why *An. coluzzii* and *An. gambiae s.s*., being anthropophilic mosquitoes, had almost all their breeding sites close to human residence within 500 m of households. In the Suba District in Kenya, households where > 90% of adult *An. gambiae s.s*. were collected also had larval sites within 300 m [58]. By breeding near blood meal hosts, female mosquitoes conserve flight energy and enable young adult progenies to easily access bloodmeals shortly after emergence.

Contrary to their choice of clean water bodies (see review by [48, 57], *An. arabiensis* larvae occurred in polluted water in drainages in Agbor-Obi. *Anopheles arabiensis* larvae had also been found in polluted urban drainages and irrigation canals in the Khartoum State of Sudan [59]. Furthermore, in Bobo-Dioulasso (Burkina Faso) where *An. arabiensis* has adapted to breeding in the polluted Houet river and can therefore transmit malaria throughout the dry season [60, 61], the vector species increased in composition among malaria vectors from 3 to 90% [60] over a period of two decades. Detections of *An. arabiensis* in these types of breeding places continue to increase [62, 63], signaling a persistent and continuous adaptation of *An. arabiensis* larvae to polluted water bodies in urban areas, thereby promoting malaria transmission throughout the dry season.

In Central Africa, larvae of *An. arabiensis* that developed in organic wastewater developed faster, resulting in adults that had longer longevity and larger phenotypic sizes, as well as increased resistance to insecticides [64]. Data on the association between *An. arabiensis* choice of breeding habitats and insecticide resistance traits are currently sparse in southern Nigeria. However, preliminary results from ongoing insecticide resistance studies in Agbor-Obi indicate the presence of the pyrethroid-resistant mutation *L995F* in larvae of *An. arabiensis* from polluted aquatic environments [unpublished data]. However, investigators are yet to find positive cases of *L995F* in *An. arabiensis* from clean water pools along stream margins in Nkwelle-Ezunaka (Anambra State).

In line with findings from the survey in southern Nigeria, a study in central Ethiopia identified *An. arabiensis* in turbid water collections [65]. Similar observations of malaria vector preference for turbid water bodies were made in Tanzania [66]. However, the species identity of mosquitoes was not determined. *Anopheles* mosquitoes typically avoid turbid for clean water for the reason that suspended insoluble particles interfere with larvae ingestion of food materials [65]. These particles also limit sunlight penetration of water and consequently, slow down the production of aquatic microphyte food materials for mosquito larvae. These may have been responsible for the avoidance of turbid water bodies by *An. gambiae s.s*. and *An. coluzzii* in southern Nigeria.

However, turbidity may have less effect on *An. arabiensis* where larvae have adequate access to food materials. In the Ye-Ebiyo et al*.* [65] study in central Ethiopia, *An. arabiensis* were unaffected by turbidity of water bodies but only when larval sites were close to flowering maize plants providing pollen grains for larvae nourishment. In the present study, turbid water bodies that contained *An. arabiensis* larvae also contained organic debris. As Jeanrenaud et al*.* [64] observed in Cameroon, these organic wastes probably served as food for *An. arabiensis* larvae in southern Nigeria.

The Asian urban malaria vector *An. stephensi* invaded Africa in 2012 [67] and has since been expanding its spatial range in the continent [68], with the most recent detections of the vector made in the West Africa sub-region [51, 69], first in 2020 in Gombe in northern Nigeria [51]. Due to its potential to drive malaria outbreaks [67, 70], *An. stephensi* surveillance has received increased attention in areas of potential invasion. Molecular analysis in the present study screened *Anopheles* larval samples for *An. stephensi* because some samples could not be identified by molecular markers used in *An. gambiae* identification; similar experiences of molecular markers failing to identify wild samples of *Anopheles* mosquitoes led to the first reports of *An. stephensi* in different locations in Africa [71]. Moreover, Sinka and colleagues [72] identified the study area in southern Nigeria among places in West Africa where ecological conditions are favourable for the invasion and establishment of *An. stephensi*.

Furthermore, given *An. stephensi* zoophagic habits [73], it is possible that the frequent pastoralists’ movement of livestock from Gombe and neighboring locations in northern Nigeria to grazing fields and slaughterhouses in southern Nigeria could provide a route for and facilitate the southward spread of *An. stephensi*. In this study, mosquito larval samplings were carried out during the wet-dry season interface at a time when *An. stephensi* occur in high abundance [72] and with a majority of larval sampling sites comprising man-made water containers where the vector species prefers to breed in urban locations [11]. Considering that the larval sampling strategy maximized opportunities for *An. stephensi* detection, the non-report of the vector among the study mosquito samples therefore suggests its absence in the sampling area and likely slow spread in the country Nigeria. However, *An. stephensi* possesses potential for rapid spatial distribution. This has been demonstrated in East Africa, where the vector species was detected in five countries within a period of 10 years [68] and in West Africa, where it was recently detected in Accra Ghana [69], just less than 3 years after the initial detection in northern Nigeria [51].

The *An. coluzzii* population in southern Nigeria was moderately genetically diverse, with a haplotype diversity index of 0.62. This suggests that vector control interventions are currently not optimally effective at reducing *An. coluzzii* abundance in the study area; otherwise, vector populations would have presented with low genetic diversity. High genetic diversity of *An. coluzzii* was attributed to variations in the ecology of larval development sites along the Gambian River in West Africa [74]. In southern Nigeria, *An. coluzzii* that developed in periurban larval sites had slightly more haplotypes than those in urban sites. However, and possibly as an indication of *An. coluzzii*’s attempt to adapt to otherwise less favorable conditions in urban ecological landscapes, one of the two dominant haplotypes detected in *An. coluzzii* occurred in close association with the urban vector population. Due to small sample size, the use of a less informative molecular marker, and the restriction of nucleotide sequencing to limited regions of the genome, it was difficult to adequately assess genetic divergence between *An*. *coluzzii* populations in the present study.

Turbidity metres or Secchi disks are recommended for reliable assessment of water turbidity; hence investigators admit that the method of assessing water turbidity based on physical observations may have been less accurate. Physical observation to assess water turbidity could also be subjective; however, in the present study, water turbidity assessment by the same person helped to address this challenge in the field. Further, the study did not systematically evaluate the effects of biological factors. Some of these biological factors, for example the presence of predators, have been shown in previous studies to affect *Anopheles* larvae in aquatic environments (reviewed in [6, 75]. However, the relative proportions of *Anopheles* larvae and the fact that sibling vectors rarely co-existed in water body sites suggest that sibling mosquitoes could be engaging in some sort of interspecific competition for resources [39, 76]). The stream margin in Nkwelle-Ezunaka (Anambra) was the only place where *An. arabiensis*, *An. gambiae s.s*. and *An. coluzzii* occurred together in a single water body. It could be that mosquito larvae, because they are less crowded in large breeding sites such as stream margins, are less likely to engage in resource competition. Still on mosquito interactions, *Anopheles* and culicine larvae had inverse associations in southern Nigeria. The inverse association observed between *Anopheles* and culicine larvae corroborates the principle that gravid dipterans typically avoid breeding places already exploited by conspecific and heterospecific females [77]. This behaviour has been reported in *An. gambiae s.s*. [78] and aims is to ensure adequate food resources for potential immature progenies and thus enhance the biological fitness of adult progenies.

[12]. 

In conclusion, the study reports different breeding habitat choices for three sibling malaria vectors in southern Nigeria. The dominant vector *An. coluzzii* prefer breeding sites in lowlands while *An. gambiae s.s*. prefer sites that are partially rather than completely exposed to sunlight. In contrast to *An. arabiensis* that display association with man-made sites outside 500 m of households, *An. coluzzii* and *An. gambiae s.s*. have a high likelihood to breed in natural sites within 500 m of households. These findings suggest that *An. coluzzii* and *An. gambiae s.s*. are more likely than *An. arabiensis* to infect humans in residential places where the vectors co-exist [79]. And as they are typically indoor feeders, *An. coluzzii* and *An. gambiae s.s*. have a greater chance of contacting and being killed by treated bed nets.

There are ongoing efforts by State governments to upscale the distribution and encourage the use of pyrethroid-treated bed nets for malaria vector control in southern Nigeria [19]. However, *An. arabiensis*, being an outdoor feeder and capable of deriving bloodmeals from multiple vertebrates in addition to humans, has a lower opportunity of encountering treated bed nets. In East Africa, treated bed nets helped control *An. gambiae s.s*., but *An. arabiensis* were only slightly affected, thus leaving behind a post-intervention phase of residual malaria transmission by *An. arabiensis* [80]. Findings of pyrethroid resistance mutations in *An. arabiensis* in the study area [unpublished data] and escalations of insecticide resistance in *An. coluzzii* in southern Nigeria [18] further dampen the prospect of vector control using treated bed nets.

Malaria control programmes in southern Nigeria could leverage findings from the present study in designing targeted larval control interventions. Across sub-Saharan Africa, larval control interventions have been explored to reduce the population abundance of *An. arabiensis* and a couple of other mosquito species that transmit infections, irrespective of vector biting location (indoors or outdoors) or insecticide resistance status (resistant or susceptible) [81, 82]. Larval control is particularly useful in the context of southern Nigeria where bed net interventions are apparently having limited effects on malaria vectors. Larval control interventions to reduce human malaria transmission are easier to implement during the dry season when several water collections providing breeding places for *Anopheles* vectors have dried up [12]. As rainfall amounts decrease, mosquito breeding activities are concentrated to fewer water collections, which, if targeted in larval control interventions, could improve the goal of reducing malaria risks in periods of little or no rainfall. The present study has identified potential sites for larval control interventions during such periods in southern Nigeria. It has also reported the absence of *An. stephensi* in selected urban and periurban locations in the area. However, southern Nigeria is exposed to *An. stephensi* invasion being a travel destination for land, air, and sea transport from places where the vector species has already established its presence. This raises a need for the National Malaria Control Programme and relevant health authorities at the subnational levels to create a system for the surveillance of urban and periurban locations for *An. stephensi. * Such surveillance should focus on man-made mosquito breeding sites where larval interventions could help to slow the spread and proliferation of *An. stephensi* in the event of an invasion.

### Supplementary Information

**Additional file 1.** Table showing the number of mosquito breeding sites positive for culicine and anopheline larvae.

**Additional file 2.** Predictors of *Anopheles* larval presence and abundance in water bodies in southern Nigeria (September to November 2022).

**Additional file 3.** Table showing the proportions of* An. coluzzii, An. gambiae ss, *and* An. arabiensis* collected at each sampling location in southern Nigeria (September to November 2022)

**Additional file 4.** Variable importance plots to illustrate the relative predictive importance of physico-chemical properties and location of mosquito breeding sites surveyed in urban and periurban areas in southern Nigeria (September to November 2022). Arranged from top to bottom in order of decreasing importance, predictors are assessed for their relative ability to determine the presence of larvae of **a** *Anopheles* species*,* **b** *An. coluzzii, ***c** *An. gambiae ss,* and **d** *An. arabiensis* in water bodies.

**Additional file 5.** Multivariate modeling of *Anopheles* larval presence and abundance/proportions in water bodies in southern Nigeria (September to November 2022).

**Additional file 6.** Median-Joining Networks of *An. coluzzii* populations in southern Nigeria. The alphabet ‘H’ next to each circle represents haplotype. The circle sizes are proportional to the haplotype frequencies. Hatched marks between haplotypes have been used to indicate the number of mutations which in each case is one mutation. The networks are arranged in the following order: **A** exposure to sunlight; **B** area (industrial & residential); **C** distance from household; **D** location (urban & periurban); **E** altitude; **F** habitat type; **G** turbidity; **H** presence of debris; **I** depth of water body; **J** pH; **K** salinity; and **L** temperature.

**Additional file 7.** Table showing nucleotide substitutions of in *Anopheles coluzzii *haplotypes in southern Nigeria, and positions of these substitutions in reference to the *An. coluzzii* Ngousso genome in VectorBase.

**Additional file 8.** Genetic analyses to assess the extent of variations between populations of *Anopheles coluzzii* mosquitoes breeding in different water bodies in southern Nigeria.

## Data Availability

All data generated and/or analysed during this study are included in this published article and in the supplementary files. The raw dataset will be made available upon reasonable request to the corresponding author. DNA sequences generated from the study have been deposited in the GenBank database using the Accession Numbers OR700033 to OR700102, and OR717035 to OR717056.

## Abbreviations

BP: Base-pair
CI: Confidence interval
DNA: Deoxyribonucleic Acid
GLM: Generalized Linear Model
*Hd*: Haplotype diversity
ITS2: Internal Transcribed Spacer-2
MCA: Multiple Correspondence Analysis
MDG: Mean Decrease Gini
OR: Odds ratio
PCA: Principal Components Analysis
PCR: Polymerase chain reaction
RF: Random Forest
SINE: Short Interspersed Elements
℃: Degree Celsius
WHO: World Health Organization
RDT: Rapid Diagnostic Test
mm: Millimeter
MJ: Median-Joining
