# Supplementary material for: Sibling species of the major malaria vector Anopheles gambiae display divergent preferences for aquatic breeding sites in southern Nigeria
Source: Malar J. 2024 Feb 27;23:60. doi: 10.1186/s12936-024-04871-9 (PMC10900747; doi:10.1186/s12936-024-04871-9)
Supplement: Supplementary file 1 — Additional file 1. Table showing the number of mosquito breeding sites positive for culicine and anopheline larvae. [file 12936_2024_4871_MOESM1_ESM.docx]

**Supplementary file 1:**

**Presence**: Culicine presence was the most important predictor of *Anopheles* larvae presence in water. This was followed by presence of debris, topographic altitude, habitat type, temperature, water exposure to sunlight, and pH, in order of decreasing importance (Random Forest Classification Accuracy 72.09%, supplementary file 2a). The odds of *Anopheles* larvae detection were greater in water bodies without debris (OR: 5.40, 95% CI: 1.49, 22.22), lowlands (OR: 4.75, 95% CI: 1.25, 21.13) and natural aquatic environments (OR: 17.42, 95% CI: 2.85, 339.61), as well as mosquito larval sites with high temperatures (OR: 6.18, 95% CI: 1.55, 31.95) and pH (OR: 5.19, 95% CI: 1.30, 26.80) (see above Table). Topographic altitude and habitat type were the only variables identified in a multivariate binomial regression model to predict presence of *Anopheles* larvae (Supplementary file 4).

**Abundance**: Overall average abundance of *Anopheles* mosquitoes was 1.82 (95% CI: 0.76, 2.89) larvae per dip. Mosquito larval habitats had greater abundance of *Anopheles* larvae if water bodies were without culicine mosquitoes (4.57, 95% CI: 1.44, 7.70 larvae per dip), were natural habitats (4.42, 95% CI: 1.32, 7.52 larvae per dip), and had shallow depths (2.15, 95% CI: 0.86, 3.44 larvae per dip) (see above Table). Furthermore, *Anopheles* larvae abundance was negatively associated with culicine larval abundance (Spearman correlation: rho = -0.49, P = 0.0008) and altitude (rho = -0.37, P = 0.014) but positively associated with temperature (rho = 0.58, P = 0.00004) and pH (rho = 0.44, P = 0.003). Habitat type and culicine presence in water were the only variables identified in a multivariate negative binomial regression model to predict *Anopheles* abundance in water bodies (Supplementary file 2).

Table showing the number of mosquito breeding sites positive for culicine and anopheline larvae

|  |  | **number of mosquito breeding sites** | | | | |
| --- | --- | --- | --- | --- | --- | --- |
|  |  | **overall** |  | **culicine positive** |  | **anopheline positive** |
| stream margin |  | 1 |  | 0 |  | 1 |
| puddle |  | 11 |  | 5 |  | 10 |
| iron pan |  | 1 |  | 1 |  | 0 |
| drum |  | 2 |  | 2 |  | 0 |
| drain |  | 21 |  | 17 |  | 11 |
| concrete well |  | 1 |  | 1 |  | 1 |
| bucket |  | 2 |  | 2 |  | 0 |
| car tire |  | 4 |  | 4 |  | 0 |
