# Supplementary material for: Sibling species of the major malaria vector Anopheles gambiae display divergent preferences for aquatic breeding sites in southern Nigeria
Source: Malar J. 2024 Feb 27;23:60. doi: 10.1186/s12936-024-04871-9 (PMC10900747; doi:10.1186/s12936-024-04871-9)
Supplement: Supplementary file 2 — Additional file 2. Predictors of Anopheles larval presence and abundance in water bodies in southern Nigeria (September to November 2022). [file 12936_2024_4871_MOESM2_ESM.docx]

**Supplementary file 2**: Predictors of *Anopheles* larval presence and abundance in water bodies in southern Nigeria (September to November 2022).

| **Predictor variables** | |  | **odds ratio** | ***P* value** |  | **mean abundance** | ***P* value** |
| --- | --- | --- | --- | --- | --- | --- | --- |
| Culicine presence | yes |  | NA | NA |  | 0.88 (0.02, 1.74) | **0.0026** |
|  | no |  |  |  |  | 4.57 (1.44, 7.70) |  |
| Household | far (intercept) |  | 0.63 (0.19, 1.87) | 0.1987 |  | 0.94 (0.00, 2.05) | 0.18 |
|  | close |  | 2.40 (0.64, 9.68) |  |  | 2.21 (0.74, 3.68) |  |
| Location | industrial (intercept) |  | 0.67 (0.17, 2.33) | 0.3337 |  | 1.20 (0.00, 2.66) | 0.45 |
|  | residential |  | 2.04 (0.49, 9.29) |  |  | 2.02 (0.67, 3.36) |  |
| Area | periurban (intercept) |  | 1.00 (0.31, 3.20) | 0.7755 |  | 2.61 (0.00, 5.75) | 0.39 |
|  | urban |  | 1.21 (0.31, 4.72) |  |  | 1.52 (0.51, 2.53) |  |
| Altitude | highland (intercept) |  | 0.40 (0.11, 1.20) | **0.0280** |  | 0.72 (0.00, 1.72) | 0.057 |
|  | lowland |  | 4.75 (1.25, 21.13) |  |  | 2.36 (0.85, 3.87) |  |
| Habitat | man-made (intercept) |  | 0.63 (0.30, 1.29) | **0.0099** |  | 0.82 (0.05, 1.59) | **0.0015** |
|  | natural |  | 17.42 (2.85, 339.61) |  |  | 4.42 (1.32, 7.52) |  |
| Turbidity | yes (intercept) |  | 1.00 (0.39, 2.56) | 0.6974 |  | 1.36 (0.00, 2.72) | 0.43 |
|  | no |  | 1.27 (0.38, 4.36) |  |  | 2.16 (0.53, 3.78) |  |
| Debris | yes (intercept) |  | 0.42 (0.13, 1.12) | **0.0133** |  | 1.03 (0.00, 2.08) | 0.16 |
|  | no |  | 5.40 (1.49, 22.22) |  |  | 2.34 (0.69, 4.00) |  |
| Vegetation | no (intercept) |  | 0.87 (0.41, 1.82) | 0.2093 |  | 1.66 (0.23, 3.10) | 0.68 |
|  | yes |  | 2.31 (0.64, 9.09) |  |  | 2.13 (0.40, 3.85) |  |
| Depth | deep (intercept) |  | 1.00 (0.24, 4.23) | 0.8266 |  | 0.40 (0.00, 0.97) | **0.049** |
|  | shallow |  | 1.19 (0.24, 5.77) |  |  | 2.15 (0.86, 3.44) |  |
| pH | low (intercept) |  | 0.71 (0.33, 1.47) | **0.0286** |  | 1.15 (0.00, 2.42) | 0.073 |
|  | high |  | 5.19 (1.30, 26.80) |  |  | 3.22 (1.21, 5.22) |  |
| Salinity | low (intercept) |  | 1.00 (0.49, 2.06) | 0.4875 |  | 1.71 (0.34, 3.07) | 0.74 |
|  | high |  | 1.60 (0.43, 6.38) |  |  | 2.10 (0.21, 3.98) |  |
| Temperature | low (intercept) |  | 0.65 (0.29, 1.37) | **0.0155** |  | 1.59 (0.10, 3.08) | 0.55 |
|  | high |  | 6.18 (1.55, 31.95) |  |  | 2.26 (0.77, 3.76) |  |
| Exposure to sunlight | partial (intercept) |  | 1.00 (0.34, 2.92) | 0.7501 |  | 2.24 (0.00, 4.92) | 0.59 |
|  | complete |  | 1.23 (0.34, 4.50) |  |  | 1.62 (0.55, 2.70) |  |

NA: Not Available due to small sample size.
