# Supplementary material for: Sibling species of the major malaria vector Anopheles gambiae display divergent preferences for aquatic breeding sites in southern Nigeria
Source: Malar J. 2024 Feb 27;23:60. doi: 10.1186/s12936-024-04871-9 (PMC10900747; doi:10.1186/s12936-024-04871-9)
Supplement: Supplementary file 3 — Additional file 3. Table showing the proportions of An. coluzzii, An. gambiae ss, and An. arabiensis collected at each sampling location in southern Nigeria (September to November 2022) [file 12936_2024_4871_MOESM3_ESM.docx]

**Supplementary file 3**: Table showing the proportions of *An. coluzzii, An. gambiae ss,* and *An. arabiensis* collected at each sampling location in southern Nigeria (September to November 2022).

| **Location** | **Number of anopheline larvae collected** | | | |
| --- | --- | --- | --- | --- |
|  | ***Anopheles*** | ***An. coluzzii*** | ***An. gambiae ss*** | ***An. arabiensis*** |
| Aduwawa | 1 | 1 | 0 | 0 |
| Agbor-Obi | 57 | 17 | 1 | 30 |
| Alihagwu | 0 | 0 | 0 | 0 |
| Boji-Boji | 30 | 50 | 1 | 0 |
| Ekenwan | 0 | 0 | 0 | 0 |
| Ekiadolor | 0 | 0 | 0 | 0 |
| GRA | 6 | 5 | 0 | 0 |
| Ibolo-Oraifite | 30 | 28 | 1 | 0 |
| Idumuoza | 8 | 8 | 0 | 0 |
| Nkpor | 1 | 2 | 9 | 0 |
| Nkwelle-Ezunaka | 115 | 16 | 26 | 1 |
| Odekpe | 66 | 47 | 1 | 0 |
| Ogbeson | 48 | 26 | 0 | 0 |
| Ogbewase | 107 | 60 | 3 | 0 |
| Onitsha | 0 | 0 | 0 | 0 |
| Owa-Alero | 39 | 12 | 0 | 0 |
| Owa-Eke | 0 | 0 | 0 | 0 |
| Owina | 42 | 25 | 0 | 0 |
| Sakponba | 0 | 0 | 0 | 0 |
| Ugbiyokho | 16 | 11 | 3 | 0 |
| Umunede | 0 | 0 | 0 | 0 |
| Uwelu | 9 | 6 | 0 | 0 |
