# Supplementary material for: Sibling species of the major malaria vector Anopheles gambiae display divergent preferences for aquatic breeding sites in southern Nigeria
Source: Malar J. 2024 Feb 27;23:60. doi: 10.1186/s12936-024-04871-9 (PMC10900747; doi:10.1186/s12936-024-04871-9)
Supplement: Supplementary file 4 — Additional file 4. Variable importance plots to illustrate the relative predictive importance of physico-chemical properties and location of mosquito breeding sites surveyed in urban and periurban areas in southern Nigeria (September to November 2022). Arranged from top to bottom in order of decreasing importance, predictors are assessed for their relative ability to determine the presence of larvae of a Anopheles species, b An. coluzzii, c An. gambiae ss, and d An. arabiensis in water bodies. [file 12936_2024_4871_MOESM4_ESM.pptx]

## Slide 1
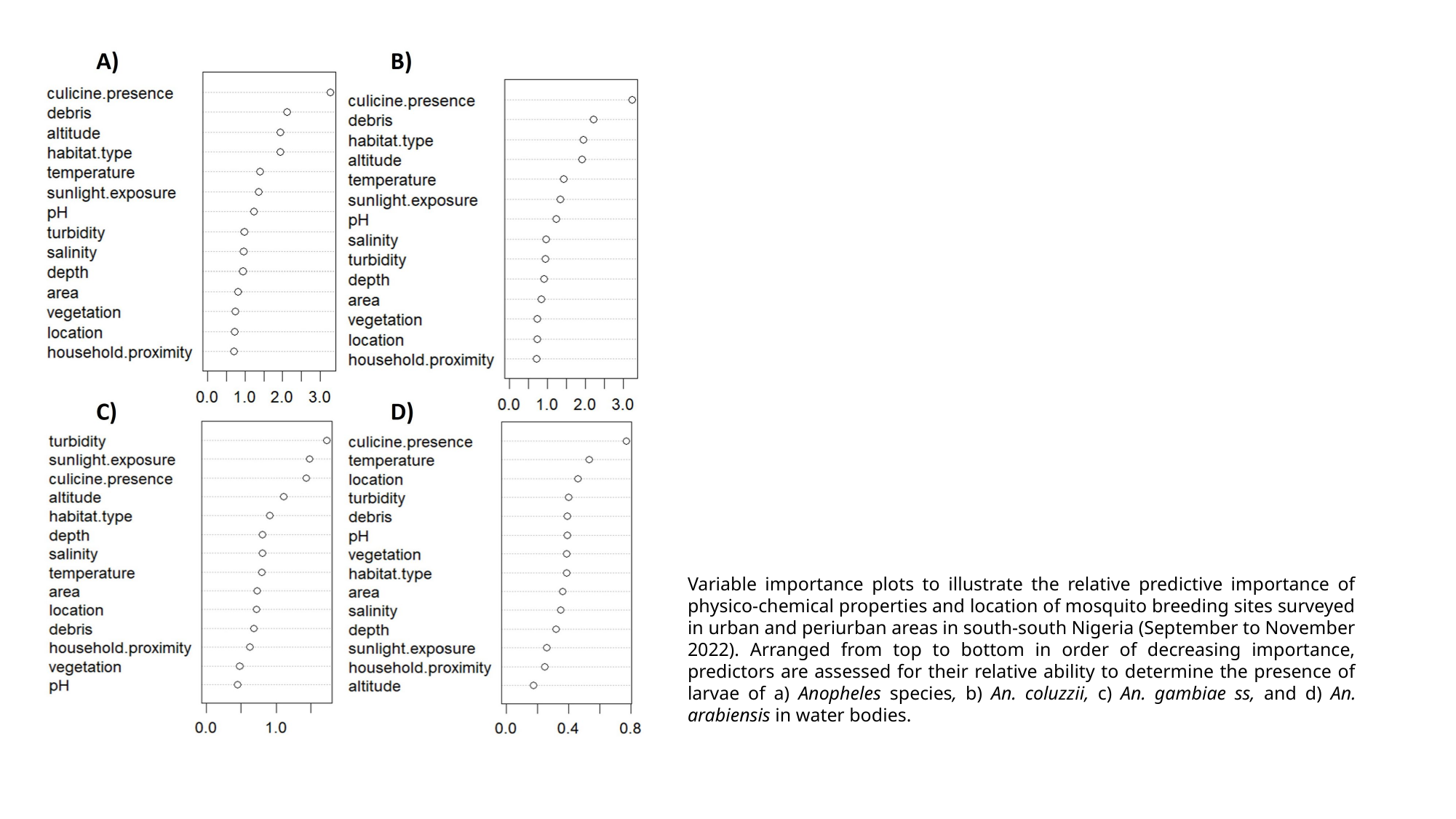

Variable importance plots to illustrate the relative predictive importance of physico-chemical properties and location of mosquito breeding sites surveyed in urban and periurban areas in south-south Nigeria (September to November 2022). Arranged from top to bottom in order of decreasing importance, predictors are assessed for their relative ability to determine the presence of larvae of a) Anopheles species, b) An. coluzzii, c) An. gambiae ss, and d) An. arabiensis in water bodies.
