# Supplementary material for: Sibling species of the major malaria vector Anopheles gambiae display divergent preferences for aquatic breeding sites in southern Nigeria
Source: Malar J. 2024 Feb 27;23:60. doi: 10.1186/s12936-024-04871-9 (PMC10900747; doi:10.1186/s12936-024-04871-9)
Supplement: Supplementary file 5 — Additional file 5. Multivariate modeling of Anopheles larval presence and abundance/proportions in water bodies in southern Nigeria (September to November 2022). [file 12936_2024_4871_MOESM5_ESM.docx]

**Supplementary file 5**: Multivariate modeling of *Anopheles* larval presence and abundance/proportions in water bodies in southern Nigeria (September to November 2022).

|  |  | ***P-*values** | | | | |
| --- | --- | --- | --- | --- | --- | --- |
|  |  | ***Anopheles* species** |  | ***An. coluzzii*** |  | ***An. gambiae ss*** |
| **Presence** |  |  |  |  |  |  |
| habitat type |  | 0.0102 |  | 0.0102 |  | 0.028 |
| altitude |  | 0.0381 |  | 0.0381 |  |  |
| exposure to sunlight |  |  |  |  |  | 0.017 |
|  |  |  |  |  |  |  |
| **Abundance / Proportions** |  |  |  |  |  |  |
| habitat type |  | 0.0315 |  |  |  |  |
| culicine presence |  | 0.0472 |  |  |  |  |
| exposure to sunlight |  |  |  |  |  | 0.0153 |
