# Supplementary material for: Sibling species of the major malaria vector Anopheles gambiae display divergent preferences for aquatic breeding sites in southern Nigeria
Source: Malar J. 2024 Feb 27;23:60. doi: 10.1186/s12936-024-04871-9 (PMC10900747; doi:10.1186/s12936-024-04871-9)
Supplement: Supplementary file 6 — Additional file 6. Median-Joining Networks of An. coluzzii populations in southern Nigeria. The alphabet ‘H’ next to each circle represents haplotype. The circle sizes are proportional to the haplotype frequencies. Hatched marks between haplotypes have been used to indicate the number of mutations which in each case is one mutation. The networks are arranged in the following order: A exposure to sunlight; B area (industrial & residential); C distance from household; D location (urban & periurban); E altitude; F habitat type; G turbidity; H presence of debris; I depth of water body; J pH; K salinity; and L temperature. [file 12936_2024_4871_MOESM6_ESM.pptx]

## Slide 1
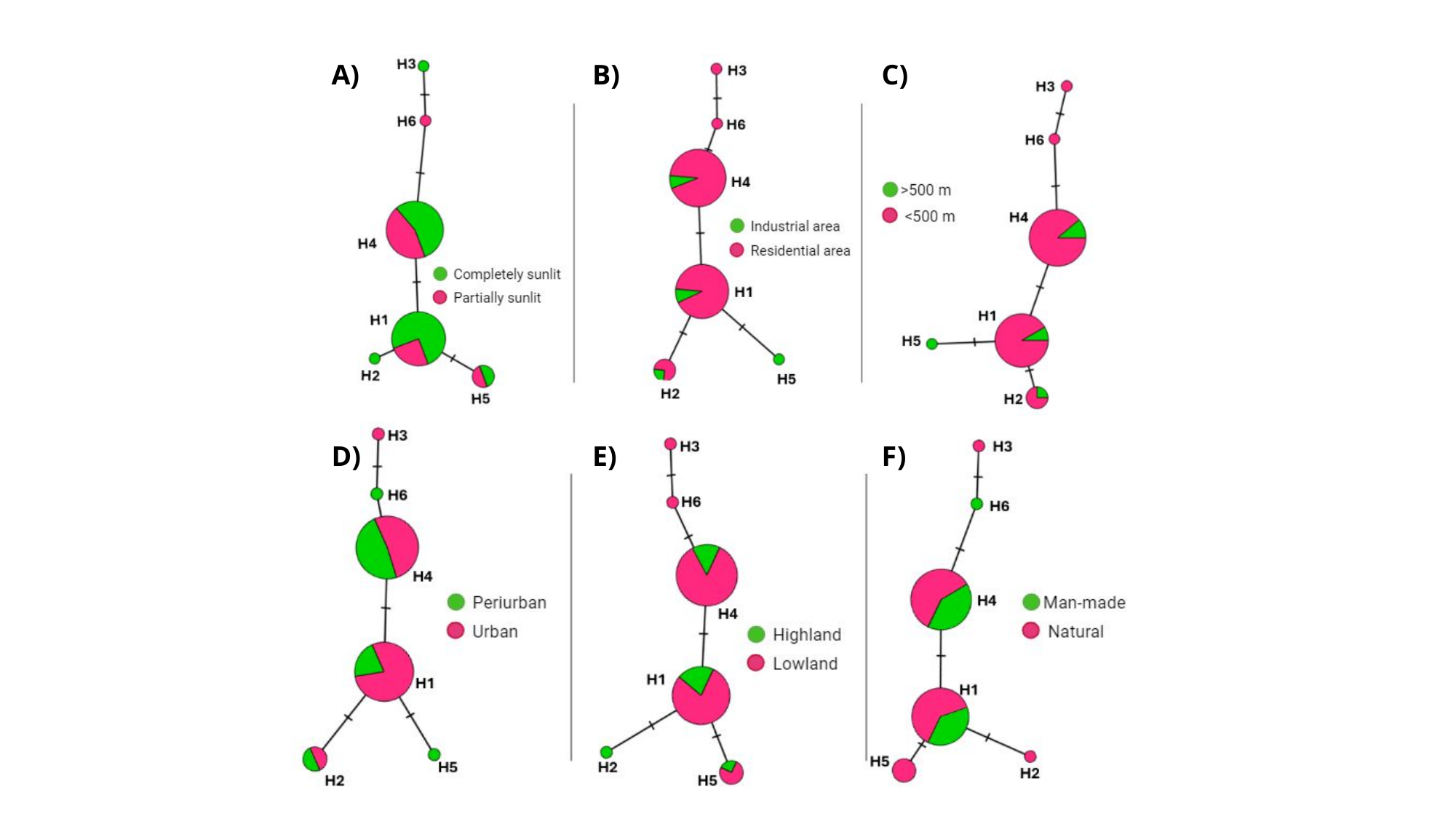

C)
A)
B)
D)
E)
F)

## Slide 2
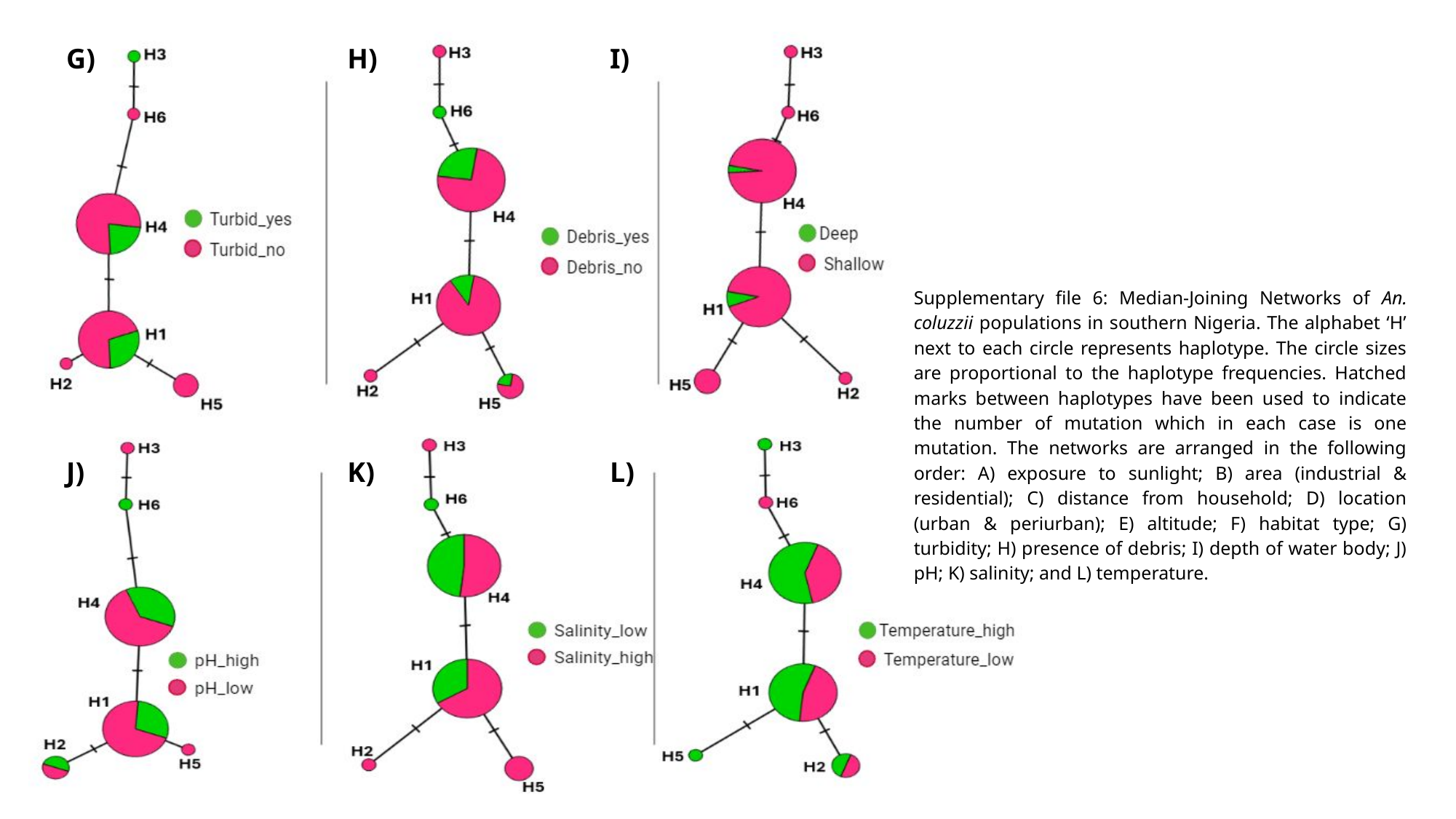

G)
H)
I)
Supplementary file 6: Median-Joining Networks of An. coluzzii populations in southern Nigeria. The alphabet ‘H’ next to each circle represents haplotype. The circle sizes are proportional to the haplotype frequencies. Hatched marks between haplotypes have been used to indicate the number of mutation which in each case is one mutation. The networks are arranged in the following order: A) exposure to sunlight; B) area (industrial & residential); C) distance from household; D) location (urban & periurban); E) altitude; F) habitat type; G) turbidity; H) presence of debris; I) depth of water body; J) pH; K) salinity; and L) temperature.
J)
K)
L)
