# Supplementary material for: Sibling species of the major malaria vector Anopheles gambiae display divergent preferences for aquatic breeding sites in southern Nigeria
Source: Malar J. 2024 Feb 27;23:60. doi: 10.1186/s12936-024-04871-9 (PMC10900747; doi:10.1186/s12936-024-04871-9)
Supplement: Supplementary file 7 — Additional file 7. Table showing nucleotide substitutions of in Anopheles coluzzii haplotypes in southern Nigeria, and positions of these substitutions in reference to the An. coluzzii Ngousso genome in VectorBase. [file 12936_2024_4871_MOESM7_ESM.docx]

Table showing nucleotide substitutions of in *Anopheles coluzzii* haplotypes in southern Nigeria, and positions of these substitutions in reference to the *An. coluzzii* Ngousso genome in VectorBase

| Haplotype ID (Number of samples) | GenBank Accession No. of representative DNA sequence | Nucleotide substitution  (nucleotide of *An. coluzzii* Ngousso > nucleotide of study sequence) | Position of nucleotide substitution in the *An. coluzzii* Ngousso genome in VectorBase |
| --- | --- | --- | --- |
|  |  |  |  |
| H1 (24) | OR700041 | A>G  G>A | 4,063,745  4,063,799 |
|  |  |  |  |
| H2 (4) | OR700046 | A>G  G>A  C>T | 4,063,745  4,063,799  4,063,769 |
|  |  |  |  |
| H3 (1) | OR700034 | C>T  A>G  A>C | 4,063,688  4,063,745  4,063,803 |
|  |  |  |  |
| H4 (27) | OR700039 | C>T  A>G  G>A | 4,063,688  4,063,745  4,063,799 |
|  |  |  |  |
| H5 (1) | OR700063 | G>A  A>G  G>A | 4,063,683  4,063,745  4,063,799 |
|  |  |  |  |
| H6 (1) | OR700087 | C>T  A>G  G>A  A>C | 4,063,688  4,063,745  4,063,799  4,063,803 |
