# Supplementary material for: Sibling species of the major malaria vector Anopheles gambiae display divergent preferences for aquatic breeding sites in southern Nigeria
Source: Malar J. 2024 Feb 27;23:60. doi: 10.1186/s12936-024-04871-9 (PMC10900747; doi:10.1186/s12936-024-04871-9)
Supplement: Supplementary file 8 — Additional file 8. Genetic analyses to assess the extent of variations between populations of Anopheles coluzzii mosquitoes breeding in different water bodies in southern Nigeria. [file 12936_2024_4871_MOESM8_ESM.docx]

Genetic analyses to assess the extent of variations between populations of *Anopheles coluzzii* mosquitoes breeding in different water bodies in southern Nigeria.

|  |  | Number of haplotypes | Number of segregating sites | *F_ST_* (Fixation Index) |
| --- | --- | --- | --- | --- |
| location | urban | 4 | 4 | 0.02938 |
|  | periurban | 5 | 4 |  |
|  |  |  |  |  |
| distance from household | >500 m (few samples) | 4 | 3 | -0.05956* |
|  | <500 m | 5 | 4 |  |
|  |  |  |  |  |
| area | industrial (few samples) | 4 | 3 | -0.03312* |
|  | residential | 5 | 4 |  |
|  |  |  |  |  |
| altitude | highland (few samples) | 4 | 3 | -0.01009* |
|  | lowland | 5 | 4 |  |
|  |  |  |  |  |
| habitat type | natural | 5 | 5 | -0.00023* |
|  | man-made (few samples) | 3 | 2 |  |
|  |  |  |  |  |
| turbidity | yes (few samples) | 3 | 3 | -0.02829* |
|  | _no | 5 | 4 |  |
|  |  |  |  |  |
| presence of debris | present | 4 | 3 | 0.00860 |
|  | absent | 5 | 5 |  |
|  |  |  |  |  |
| water depth | deep (few samples) | 2 | 1 | -0.12357* |
|  | shallow | 6 | 5 |  |
|  |  |  |  |  |
| pH | high | 5 | 5 | -0.02647* |
|  | low (few samples) | 4 | 3 |  |
|  |  |  |  |  |
| salinity | high | 3 | 2 | 0.04907 |
|  | low | 5 | 5 |  |
|  |  |  |  |  |
| temperature | low (few samples) | 4 | 3 | -0.03256* |
|  | high | 5 | 5 |  |
|  |  |  |  |  |
| exposure to sunlight | partially sunlit | 4 | 3 | 0.01230 |
|  | completely sunlit | 5 | 5 |  |

*Estimate is less reliable due to small sample size
